# An Arabidopsis downy mildew non-RxLR effector suppresses induced plant cell death to promote biotroph infection

**DOI:** 10.1093/jxb/eraa472

**Published:** 2020-10-16

**Authors:** Florian Dunker, Lorenz Oberkofler, Bernhard Lederer, Adriana Trutzenberg, Arne Weiberg

**Affiliations:** 1 Faculty of Biology, Genetics, Biocenter Martinsried, LMU Munich, Planegg-Martinsried, Germany; 2 Swedish University of Agricultural Sciences, Sweden

**Keywords:** *Arabidopsis thaliana*, downy mildew, host-induced gene silencing (HIGS), *Hyaloperonospora arabidopsidis*, non-RxLR cysteine-rich protein effectors (CRs), obligate biotrophic plant parasite

## Abstract

Our understanding of obligate biotrophic pathogens is limited by lack of knowledge concerning the molecular function of virulence factors. We established Arabidopsis host-induced gene silencing (HIGS) to explore gene functions of *Hyaloperonospora arabidopsidis*, including *CYSTEINE-RICH PROTEIN* (*HaCR*)*1*, a potential secreted effector gene of this obligate biotrophic pathogen. *Ha*CR1 HIGS resulted in *H. arabidopsidis*-induced local plant cell death and reduced pathogen reproduction. We functionally characterized *Ha*CR1 by ectopic expression in *Nicotiana benthamiana*. *Ha*CR1 was capable of inhibiting effector-triggered plant cell death. Consistent with this, *Ha*CR1 expression in *N. benthamiana* led to stronger disease symptoms caused by the hemibiotrophic oomycete pathogen *Phytophthora capsici*, but reduced disease symptoms caused by the necrotrophic fungal pathogen *Botrytis cinerea*. Expressing *Ha*CR1 in transgenic Arabidopsis confirmed higher susceptibility to *H. arabidopsidis* and to the bacterial hemibiotrophic pathogen *Pseudomonas syringae*. Increased *H. arabidopsidis* infection was in accordance with reduced *PATHOGENESIS RELATED* (*PR*)*1* induction. Expression of full-length *Ha*CR1 was required for its function, which was lost if the signal peptide was deleted, suggesting its site of action in the plant apoplast. This study provides phytopathological and molecular evidence for the importance of this widespread, but largely unexplored class of non-RxLR effectors in biotrophic oomycetes.

## Teaser

Induced cell death is a crucial component of plant defence. An extracellular downy mildew cysteine-rich protein effector inhibited plant cell death enabling host colonization.

## Introduction

Oomycetes include some notorious plant pathogens that severely reduce global crop yield and cause enormous economic loss every year. To date, oomycete pest management relies on inbreeding of *RESISTANCE* (*R*) genes and chemical plant protection. In this context, the occurrence of new virulent pathogen genotypes that overcome *R* gene-mediated resistance or chemical crop protection jeopardizes food security (Fry, 2008; Cohen *et al.*, 2015; Delmas *et al.*, 2017). Thus, there is an urgent need for developing innovative, sustainable strategies to control oomycete pests. However, a lack of understanding of pathogen virulence at the molecular level restricts this goal.

In oomycetes, classical forward or reverse genetics approaches remain challenging due to di- or polyploidy, and due to the fact that many oomycetes are obligate biotrophs, like the downy mildew pathogen *Hyaloperonospora arabidopsidis* infecting the model plant Arabidopsis (Coates and Beynon, 2010). Obligate biotrophs are impossible to grow in axenic culture despite numerous attempts (McDowell, 2014), and thus genetic transformation of these organisms is not achievable. Alternative approaches to investigating the function of pathogen effectors and other types of virulence genes in obligate biotrophs, which do not rely on pathogen transformation, are therefore highly appreciated. *Hyaloperonospora arabidopsidis* is one of the most used model pathogens to investigate Arabidopsis innate immune response to obligate biotrophs and was ranked the second most important oomycete pathogen by researchers in terms of scientific and economic relevance (Kamoun *et al.*, 2015). It is highly adapted and specialized to its sole natural host plant, Arabidopsis, and infection frequently occurs in wild Arabidopsis plants (McDowell, 2014; Agler *et al.*, 2016).

Genome sequencing of *H. arabidopsidis* uncovered a large repertoire of over 100 putative effector genes suggesting an extensive resource to suppress plant immunity (Baxter *et al.*, 2010) and to enable host cell reprogramming for pathogen accommodation and propagation (Thordal-Christensen *et al.*, 2018). Oomycete effectors are typically classified by sequence features into RxLRs, Crinklers, necrosis-inducing like proteins, elicitins, and if no further sequence homology is apparent, cysteine-rich (CR) proteins (Cabral *et al.*, 2011). Current research in oomycete effectors focuses on RxLRs that are typically translocated into host cells and are relatively easy to predict *in silico* (Anderson *et al.*, 2015). *Hyaloperonospora arabidopsidis* probably employs RxLRs to modulate plant immunity, too (Fabro *et al.*, 2011; Pel *et al.*, 2014). Nevertheless, a defined molecular function of only just a few oomycete effectors has been reported, mainly through ectopic expression *in planta* (Caillaud *et al.*, 2013; Wirthmueller *et al.*, 2018). In addition, non-RxLR effectors presumably contribute to virulence, as well. Nevertheless, *H. arabidopsidis* non-RxLR CR protein effectors remain functionally uncharacterized, despite the fact that they comprise some of the most highly expressed *H. arabidopsidis* genes during infection (Cabral *et al.*, 2011; Asai *et al.*, 2014).

Artificial expression of double-stranded RNA (dsRNA) in host plants can lead to silencing of complementary genes in their pathogens and pests, a strategy known as host-induced gene silencing (HIGS) (Baum *et al.*, 2007; Mao *et al.*, 2007; Koch and Kogel, 2014). Indeed, HIGS is a powerful method of choice for reverse genetics in plant-associated organisms with no transformation protocols available, such as root knot nematodes, mycorrhizal fungi and biotrophic pathogens, like powdery mildew and rust fungi (Nowara *et al.*, 2010; Helber *et al.*, 2011; Pliego *et al.*, 2013; Dinh *et al.*, 2014; Yin and Hulbert, 2018). Regarding oomycetes, an initial HIGS approach in Arabidopsis failed to knockdown gene expression of *Phytophthora parasitica* although HIGS small interfering RNA accumulated in the plant (Zhang *et al.*, 2011). Nevertheless, HIGS was successfully introduced in *Solanum tuberosum* (potato) against the hemibiotrophic pathogen *Phytophthora infestans* and in lettuce against the downy mildew pathogen *Bremia lactucae*, conferring plant disease resistance (Govindarajulu *et al.*, 2015; Jahan *et al.*, 2015). Conversely, silencing of the RxLR-type avirulence gene *Avr3a1* by HIGS allowed infection of resistant tobacco by *Phytophthora capsici* (Vega-Arreguín *et al.*, 2014), highlighting the power of HIGS to enable functional gene studies in plant–oomycete interactions. Recently, HIGS was suggested to induce gene suppression of infecting fungal and oomycete pathogens by plant endogenous small RNAs in Arabidopsis and in cotton, proposing a novel RNA-based plant defence mechanism (Zhang *et al.*, 2016; Cai *et al.*, 2018; Hou *et al.*, 2019; Hou and Ma, 2020). In this report, we used Arabidopsis HIGS for targeted gene knockdown of the *H. arabidopsidis CYSTEINE-RICH* (*HaCR*)*1* and ectopic plant expression of *Ha*CR1 to explore the function of this non-RxLR CR effector protein in plant–pathogen interactions.

## Material and methods

### Plant materials and cultivation

Arabidopsis wild type (WT) Col-0, HIGS construct transformants and *Hyaloperonospora arabidopsidis Ha*CR1 overexpression lines were cultivated under long day conditions in a growth chamber (16 h light: 8 h dark, 22 °C and 150 µmol m^−2^ s^−1^ photon flux density). Fourteen-day-old seedlings were used for *H. arabidopsidis* inoculation.

Arabidopsis effector overexpression lines were cultivated under short day conditions in a walk-in growth chamber (8 h light: 16 h dark, 22 °C and 150 µmol m^−2^ s^−1^ photon flux density). Five- to six-week-old plants were used for bacterial inoculation.

Wild tobacco (*Nicotiana benthamiana* Domin) plants were grown in a walk-in growth chamber under long day conditions (16 h light: 8 h dark, 22 °C and 275 µmol m^−2^ s^−1^ photon flux density) for 4 weeks prior to *Agrobacterium tumefaciens*-mediated transformation.

### Microorganism cultivation

*Hyaloperonospora arabidopsidis* Gäum. strain Noco2 was maintained on Arabidopsis Col-0 seedlings and used for plant inoculation at a concentration of 2–2.5×10^4^ spores ml^−1^, as described previously (Ried *et al.*, 2019). *Phytophthora capsici* Leonian strain LT263 (Hurtado-Gonzales and Lamour, 2009) was cultured on rye agar plates (Caten and Jinks, 1968) for 3 d at room temperature before plant inoculation. *Botrytis cinerea* Pers. strain B05.10 was cultured on HA agar plates for 2 d prior to plant inoculation. *Pseudomonas syringae* pv. *tomato* Van Hall (*Pst*) DC3000 and *Pst* DC3000 *hrcC*^*−*^ mutant were cultured on LB agar plates with rifampicin.

### Plasmid construction

For HIGS constructs targeting *HaCR1*, *HaACT A*, *HaA1E*, or *HaDCL1*, target gene fragments of 334, 311, 267, and 256 bp length, respectively, were amplified from cDNA using home-made Phusion DNA polymerase. The DNA stretches were tested for off-targets in Arabidopsis and *H. arabidopsidis* cDNAs using the Si-Fi2.1 tool (http://labtools.ipk-gatersleben.de/index.html) and have a maximum of two off-target small RNAs, as opposed to hundreds of effective on-target small RNAs.

RNA hairpins were cloned under the control of the strong *proLjUBI* promoter using the previously described and validated Golden Gate based RNAi plasmid assembly kit, containing the Arabidopsis *AtWRKY33* intron 1 and the 35S terminator (Binder *et al.*, 2014). Yellow fluorescent protein (YFP; mCherry for green fluorescent protein (GFP)-RNAi hairpin) was used in the final expression vector as an *in planta* transformation marker, and *Agrobacterium tumefaciens* AGL1 was transformed with completed vector constructs via electroporation.

Plasmid constructs for *in planta* expression were also made using the plant Golden Gate plasmid assembly kit (Binder *et al.*, 2014). The coding sequence of *HaCR1* was obtained by PCR amplification of *H. arabidopsidis* cDNA with Phusion High-Fidelity Polymerase (New England Biolabs, Frankfurt, Germany). The *HaCR1* coding sequence lacking the signal peptide was amplified with home-made Taq DNA polymerase, as Phusion polymerase did not result in any amplification. Taq amplicons were blunted using Phusion DNA polymerase. All PCR products were validated by Sanger sequencing (LMU Genomics service unit, Planegg, Germany) before expression vector assembly.

The binary expression vector was assembled by ligation of the C-terminal *GFP*-tagged full-length or signal peptide-deleted *HaCR1* sequences under the control of the *proLjUBI* promoter. As a control, a vector expressing only *GFP* was constructed. A list of primers used for the construction of plasmids is provided in Supplementary Table S1 at *JXB* online.

### Arabidopsis transformation

Arabidopsis Col-0 plants were transformed by the floral dip method with *A. tumefaciens* strain AGL1, as described previously (Clough and Bent, 1998). Transformants from effector overexpression experiments were selected by kanamycin resistance on ½ MS agar plates with 1% sucrose and 50 mg l^−1^ kanamycin, as described previously (Harrison *et al.*, 2006). Transformants expressing HIGS constructs were selected at the seedling stage by YFP fluorescence using a fluorescence stereo microscope. All experiments were performed on transgenic Arabidopsis plants in the T_2_ generation.

### Trypan Blue staining

Infected leaves were stained to visualize oomycete infection structures with Trypan Blue (Sigma-Aldrich, Steinheim, Germany), as previously described (Koch and Slusarenko, 1990). Leaves were de-stained with saturated chloralhydrate (Sigma-Aldrich) and imaged on a CTR 6000 microscope (Leica Microsystems, Wetzlar, Germany) with a DFC450 CCD-Camera (Leica).

### RNA isolation, cDNA synthesis, and quantitative PCR

For DNA or RNA analysis, five Arabidopsis leaves from infected plants were pooled into one biological replicate, frozen in liquid nitrogen, and ground to powder using steel beads and a bead mill (MM400, Retsch, Haan, Germany). RNA was isolated using a modified cetyltrimethylammonium bromide-based protocol (Bemm *et al.*, 2016). DNA digestion was performed on 1 µg total RNA using RNAse-free DNAse I (Thermo Fisher Scientific, Vilnius, Lithuania) after the manufacturer’s instructions. For cDNA synthesis, SuperScript III (Thermo Fisher Scientific) and oligo-dT primers (50 µM) were used, following the manufacturer’s instructions. Gene expression was determined by quantitative PCR (qPCR) using the EvaGreen master mix (Metabion, Planegg, Germany) or primaQUANT SYBRGreen Mastermix (Steinbrenner Laborsysteme, Wiesenbach, Germany) and a qPCR cycler (QuantStudio5, Thermo Fisher Scientific). For normalization of quantification values, *H. arabidopsidis ELONGATION FACTOR 1α* (*HaEF1α*) was validated as a reference gene using *40S ribosomal protein S3A* (*HaWS021*) and *β-TUBULIN* (*HaTUB*) genes (Yan and Liou, 2006) (see Supplementary Fig. S1). For expression analysis of Arabidopsis genes, *AtACTIN2* (*AtACT2*) was used as reference gene (An *et al.*, 1996). Stable expression of *AtACT2* was validated by correlating with the expression of *AtTUBULIN* (*AtTUB*). *AtTUB* was used in combination with *AtUBQ10* as reference genes when was *AtACT2* subjected for gene expression analysis itself. Differential expression was calculated using the 2^−ΔΔ*C*t^method (Livak and Schmittgen, 2001) and the reference gene(s) used for normalization are detailed in the figure legends. All primers with annealing temperature are listed in Supplementary Table S1.

### Phylogenetic analysis

Conserved protein domains and motifs were analysed with InterPro (https://www.ebi.ac.uk/interpro/). Sequences of group I and II CR proteins from *H. arabidopsidis* were obtained from the NCBI GenBank (accession numbers JF800102-JF800110). The draft genome sequence of the Noco2 single spore isolate Noks1 was obtained from NCBI GenBank (accession number PRJNA298674). A phylogenetic tree and sequence alignment were constructed with CLC Main Workbench 7.6.4 (https://digitalinsights.qiagen.com/), with default settings for the alignment, and the tree was constructed using neighbour joining and Jukes–Cantor distance measurement. A cysteine-rich protein from *Phytophthora parasitica* (PpCR; F443_03861) was used to root the tree.

### Transient *Nicotiana benthamiana* transformation

*Agrobacterium tumefaciens* strain AGL1 was grown for 2 d at 28 °C in LB medium with appropriate antibiotics. Bacteria were harvested by centrifugation at 4000 *g* and incubated in induction buffer (10 mM MES–KOH pH 5.6, 10 mM MgCl_2_, 150 µM acetosyringone) for 1–2 h. The OD_600_ was adjusted to 0.5 for each construct to perform pathogen assays and 0.25 for protein localization experiments. Leaves were infiltrated using needleless syringes and plants were replaced in the growth chamber under the same conditions.

### *Phytophthora*/*Botrytis* pathogen assay

Two days after *A. tumefaciens* infiltration, *N. benthamiana* plants were inoculated with the respective pathogen by adding two Ø 0.5 cm agar plugs with mycelium per leaf. Images were taken with a camera and lesion sizes were measured with Fiji/ImageJ software (https://imagej.net/Fiji).

### Cell death suppression assay

*HaCR1-GFP*, *∆SP-HaCR1-GFP*, or *GFP* plasmids were co-transformed with the effector *AvrE1* cloned from *Pseudomonas syringae* pv. *tomato* that elicits cell death in *N. benthamiana* (Badel *et al.*, 2006). *Agrobacterium tumefaciens* cell concentration of all constructs was equally adjusted to a final OD_600_ of 1.0. Infiltration was performed on 4-week-old *N. benthamiana* plants. Each individual construct was injected into the same leaf at separate areas (1.5 cm^2^). Pictures of the leaves were taken 5 d post-infiltration and analysed by mean grey value counts using the Fiji/ImageJ software (https://imagej.net/Fiji).

### Epifluorescence and confocal microscopy

Overview pictures of *N. benthamiana* leaves were taken using a M165 FC epifluorescence stereomicroscope (Leica microsystems) with a GFP/DsRED filter. Confocal laser-scanning microscopy of *N. benthamiana* leaves was performed with an upright SP5 confocal laser scanning microscope (Leica Microsystems) and imaged using an HCX IRAPO L256/0.95W objective (Leica Microsystems). For image acquisition, the resolution was set to 1024×1024 pixels and the frame average to 4. Fluorescent tags were excited using an argon laser at 20% power. GFP was excited with a 488 nm laser line and detected at 500–530 nm, cyan fluorescent protein (CFP) was excited with a 458 nm laser line and detected at 465–505 nm.

### Collection of apoplastic wash fluid and apoplastic protein isolation

Six-week-old *N. benthamiana* plants were transformed with *A. tumefaciens*, as described above. To isolate apoplastic wash fluids we adapted and modified a published protocol for the isolation of apoplastic fluids and vesicles from Arabidopsis (Rutter *et al.*, 2017), describing here the modifications. Two days after infiltration, the leaves were detached and the leaf surface was gently washed with ultrapure water. The leaflet was cut along the midrib and damaged areas were excised. The leaf stripes were washed in ultrapure water for 5 min to remove cytoplasm contamination from the cut surface. The leaf pieces were vacuum infiltrated with apoplastic wash buffer (20 mM MES, 2 mM CaCl_2_, 0.1 M NaCl, pH 6.0 with NaOH) for 4 min with a desiccator and the vacuum slowly removed within 4 min. The apoplastic fluid was then collected via centrifugation for 15 min at 250 *g* and 4°C. The isolated apoplastic wash fluid was split and one part directly used for apoplastic protease activity measurement. The other part was used for total apoplastic protein extraction. Therefore, proteins were collected by trichloroacetic acid and acetone precipitation and dissolved in 5× protein loading dye (225 mM Tris–HCl pH 6.8, 450 mM dithiothreitol (DTT), 5% SDS, 50% glycerol, 0.05% Bromphenol Blue).

### Total protein extraction and western blot analysis

Proteins were extracted from *N. benthamiana* leaf discs, as described previously (Cerri *et al.*, 2017). Protein extracts were supplemented with 5× loading dye (225 mM Tris–HCl pH 6.8, 450 mM DTT, 5% SDS, 50% glycerol, 0.05% Bromphenol Blue), boiled for 5 min at 95 °C, and separated via SDS-PAGE. Transgene constructs were detected via western blot using α-GFP antibody (Clones 7.1 and 13.1; Roche Diagnostics, Mannheim, Germany) and by secondary antibody α-mouse IRDye800 (Li-Cor, Bad Homburg, Germany). The membrane was scanned with the Odyssey imaging system (Li-Cor). To visualize total protein content, either the polyacrylamide gel was stained using silver nitrate (Roti-Black P, Carl Roth, Karlsruhe, Germany) or the membrane after blotting was stained with staining solution (0.1% Coomassie Brilliant Blue G250 (Serva, Heidelberg, Germany), 10% acetic acid, 40% ethanol in water) and de-stained with a solution of 10% acetic acid–30% ethanol.

### Plant protease activity assay

Plant protease activity of isolated apoplastic wash fluid was determined using the Pierce fluorescent protease assay kit (Thermo Fisher Scientific) following the manufacturer’s instructions for samples with low pH. Fluorescence was determined using a microplate reader (Tecan, Männedorf, Switzerland) with excitation and emission wavelengths of 485 nm and 538 nm, respectively. Protease activity was normalized on total protein content determined by Coomassie Brilliant Blue staining and quantified using Fiji/ImageJ (https://imagej.net/Fiji) as previously described (Miller, 2010).

### *Pseudomonas* syringae pathogen assay

Liquid LB medium with rifampicin was inoculated with a single colony of *Pst* DC3000 and *Pst* DC3000 *hrcC*^*−*^ and incubated overnight at 28°C. The bacteria were collected by centrifugation and diluted with 10 mM MgCl_2_ to a final OD_600_ of 0.0006. Leaves of 5- to 6-week-old Arabidopsis plants grown under short day conditions were infiltrated with the bacterial suspension, covered with a transparent lid and incubated under long day conditions. Two to three days after inoculation, leaf chlorosis of infiltrated leaves became visible and three leaf discs per biological replicate were harvested with a cork borer (Ø 0.6 cm). Leaf discs were homogenized in 10 mM MgCl_2_ using a bead mill (MM400, Retsch) and two steel beads. A dilution series was plated on LB plates with rifampicin, and colony forming units were counted using a stereomicroscope.

## Results

### HIGS is a powerful tool for functional gene studies in *H. arabidopsidis*

**Fig. 1.**
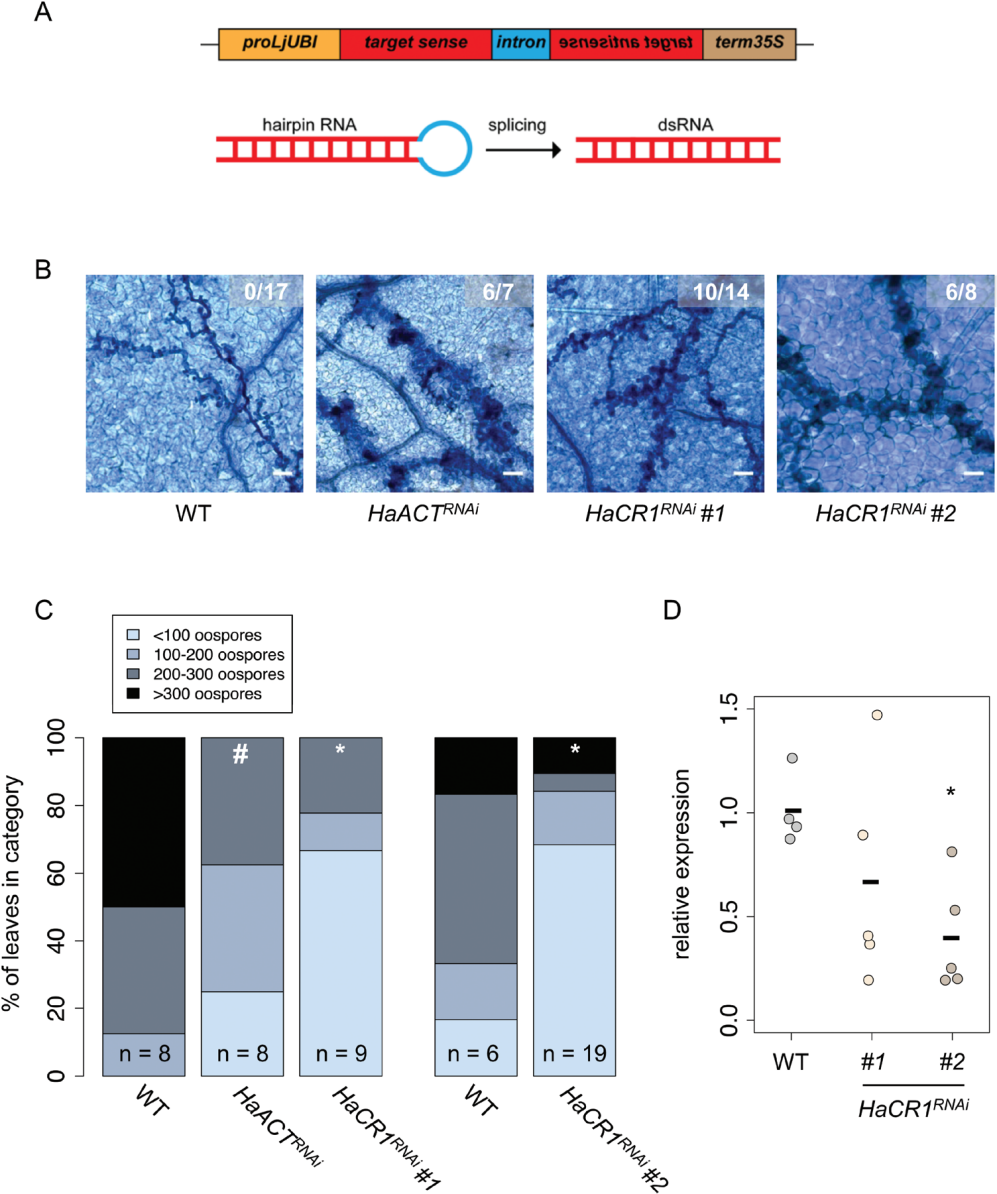
Targeted gene knockdown of *HaCR1* and *HaACT A* via HIGS in Arabidopsis. (A) Representative scheme of HIGS constructs. (B) Trypan Blue staining of *H. arabidopsidis*-infected *HaACT*^*RNAi*^ and *HaCR1*^*RNAi*^ plants at 7 dpi revealed induced trailing necrosis around the pathogen hyphae. At minimum, seven leaves were inspected per genotype, from which a representative image is shown. Numbers in micrographs represent observed leaves with trailing necrosis per total inspected leaves. Scale bars represent 50 µm. (C) *HaACT*^*RNAi*^ and *HaCR1*^*RNAi*^ plants allowed lower numbers of *H. arabidopsidis* oospore production compared with WT plants at 7 dpi. Oospore density (in categories) was counted with *n* representing the number of inspected leaves. *P≤0.05, #*P*≤0.1, significant difference by χ ^2^ test. (D) *HaCR1* gene knockdown in *H. arabidopsidis* was quantified by qRT-PCR in two independent *HaCR1*^*RNAi*^ lines upon infection at 4 dpi, with WT as control plants and *HaEF1α* and *HaWS021* as reference genes. The bars indicate the average of at least three biological replicates each comprising six to eight plant leaves. *P≤0.05, significant difference by Student’s *t*-test.

We used Arabidopsis HIGS in order to investigate the functional roles of genes in the obligate biotrophic plant pathogen *H. arabidopsidis*. As proof of concept, we chose four *H. arabidopsidis* candidate genes as HIGS targets, for which we presumed that gene knockdown would affect pathogen infection, namely the housekeeping gene *ACTIN A* (*HaACT*^*RNAi*^), the *CYSTEINE-RICH1* protein gene (*HaCR1*^*RNAi*^), an *ALDOSE-1-EPIMERASE* (*HaA1E*^*RNAi*^) gene, and the type-III RNA endonuclease gene *DICER-LIKE1* (*HaDCL1*^*RNAi*^). *HaACT A* (*HpaG807716*) is constitutively expressed in *H. arabidopsidis* and other oomycetes, and is a crucial component of the cytoskeleton (Ketelaar *et al.*, 2012). *Ha*CR1 (*HpaG806256*) and *Ha*A1E (*HpaG814621*) are putative pathogenicity factors that are highly expressed in *H. arabidopsidis* during Arabidopsis infection (Asai *et al.*, 2014). *Ha*DCL1 (*HpaG808216*) is likely involved in biogenesis of *H. arabidopsidis* small RNAs, which we recently found to play an important role in suppressing plant genes for host infection (Dunker *et al.*, 2020). The fungal plant pathogen *Botrytis cinerea* uses small RNAs for Arabidopsis plant infection, too (Weiberg *et al.*, 2013), and HIGS against *Botrytis DCL*s indeed conferred disease resistance (Wang *et al.*, 2016). To clone HIGS RNA hairpin transgenes (Fig. 1A), we chose target gene fragments that we predicted to not induce any off-target silencing either in *H. arabidopsidis* or in Arabidopsis using the Si-Fi2.1 tool (Lück *et al.*, 2019). We confirmed the overall efficiency of our generated hairpin constructs by transient expression of a *GFP* RNA hairpin in leaves of the *N. benthamiana* line 16c stably expressing GFP (Ruiz *et al.*, 1998) by *A. tumefaciens* infiltration. Transgenic *GFP* expression was clearly suppressed at local *A. tumefaciens* infiltration zones, as previously described (Kościańska *et al.*, 2005), and release of repression by infiltration of a construct to co-express the viral RNAi suppressor protein p19 (Silhavy *et al.*, 2002) verified *GFP* silencing via RNAi (see Supplementary Fig. S2). Therefore, we concluded that our constructs would effectively confer RNA silencing. Hence, we generated stable transgenic Arabidopsis lines expressing HIGS RNA hairpins in the ecotype Col-0. T_2_ plants were selected and inoculated with the *H. arabidopsidis* isolate Noco2, which is virulent on Arabidopsis Col-0. We inspected infection phenotypes of WT and HIGS plants at 4 and 7 d post-inoculation (dpi) by light microscopy using the Trypan Blue staining method. Pathogen hyphae and haustoria were visible in all plant lines at 4 dpi, confirming successful infection (Supplementary Fig. S3). At 7 dpi, local plant cell death was visible around the infecting hyphae in plants of a *HaACT*^*RNAi*^ and two independent *HaCR1*^*RNAi*^ lines (Fig. 1B). Such *H. arabidopsidis*-induced local plant cell death, known as trailing necrosis, is associated with enhanced disease resistance against *H. arabidopsidis* infection (Uknes *et al.*, 1992). Trailing necrosis also occurred, albeit to a lesser extent, in *HaA1E*^*RNAi*^ plants (Supplementary Fig. S4A), but not in WT (Fig. 1C) or in *HaDCL1*^*RNAi*^ plants (Supplementary Fig. S4B). In *HaACT*^*RNAi*^ and *HaCR1*^*RNAi*^ plants, trailing necrosis was accompanied by a reduction of *H. arabidopsidis* oospore production (Fig. 1C; Supplementary Fig. S5). To examine the effect of HIGS on target gene expression, we determined transcript levels of *HaACT A* and *HaCR1* in WT and *HaACT*^*RNAi*^ or *HaCR1*^*RNAi*^ plants, respectively. We did not detect any target gene amplification by RT-PCR in non-inoculated HIGS plants, ensuring that the target gene-specific primers did not produce any signal derived from the HIGS hairpin construct (Supplementary Fig. S6). Stable expression of the reference gene *ELONGATION FACTOR 1α* (*HaEF1α*, *HpaG809424*) was validated by quantitative reverse transcription (qRT)-PCR correlating their *C*_t_ values with two other reference genes, *40S ribosomal protein S3A* (*HaWS021*, *HpaG810967*) and *HaTUB* (a β-tubulin, *HpaG814031*), and of *AtACt2* (*At3G18780*) by plotting the *C*_t_ values against *AtTUB* (*At5G62690*) (Supplementary Fig. S1A–D) according to the MIQE guidelines (Bustin *et al.*, 2009). Gene silencing of *HaACT A* and *HaCR1* was evident at 4 dpi in a *HaACT*^*RNAi*^ line (Supplementary Fig. S7A) and in two independent HIGS lines of *HaCR1*^*RNAi*^ (Fig. 1D). Neither target gene was suppressed at 7 dpi. (Supplementary Fig. S7B). The *HaACT*^*RNAi*^ plants appeared smaller than WT plants (Supplementary Fig. S8A), and thus we assumed an off-target effect on Arabidopsis *ACTIN* by the *HaACT*^*RNAi*^ transgene. We determined the expression of the two Arabidopsis *ACTIN* genes, *AtACT2* (*At3G18780*) and *AtACT11* (*At3G12110*), showing the highest sequence similarity to *HaACT A*. The qRT-PCR analysis did not indicate any significant down-regulation of *AtACT2* or *AtACT11* upon *H. arabidopsidis* infection at 4 dpi (Supplementary Fig. S8B), rendering the connection between the HIGS construct and the plant growth phenotype unclear. We considered the possibility that such a growth phenotype in *HaACT*^*RNAi*^ plants could have influenced the infection outcome with *H. arabidopsidis*. The transgenic Arabidopsis *HaCR1*^*RNAi*^ plants did not display any obvious pleiotropic effects, and we concluded that pathogen-induced plant cell death and enhanced disease resistance were due to *HaCR1* silencing. With these data, we considered that *Ha*CR1 was an important virulence factor of *H. arabidopsidis* to infect Arabidopsis.

### *Ha*CR1 is a member of the *H. arabidopsidis* CR effector protein family

To seek the potential function of *Ha*CR1, we performed *in silico* protein sequence analysis. The *Ha*CR1 172-amino-acid sequence has a predicted 19-amino-acid secretion signal peptide, but no further predicted functional domains or motifs. Sixteen family members of the *Ha*CR proteins were previously classified into group I and group II by their cysteine pattern, with *Ha*CR1 belonging to group I (Cabral *et al.*, 2011). We accomplished phylogenetic analysis on the group I and II *Ha*CR proteins using a *Phytophthora capsici* CR protein to root the phylogenetic tree. Phylogeny analysis suggested separate clades of *Ha*CRs, with *Ha*CR1 forming one branch with its close homologues *Ha*CR3 (*HpaG813024*) and *Ha*CR4 (*HpaG806254*), and the second clade consisting in *Ha*CR5 (HpaG814422), *Ha*CR6 (Cabral *et al.*, 2011), and *Ha*CR7 (*HpaG814216*). Further *Ha*CR clades were not explicitly reliable due to overall low sequence conservation (see Supplementary Fig. S9A). We did not detect *Ha*CR2, a *HaCR1* homologue that was previously reported in the *H. arabidopsidis* strain Waco9 (Cabral *et al.*, 2011), in the genome sequence of Noks1, a single-spore isolate of Noco2 (Bailey *et al.*, 2011). Waco9 *Ha*CR2 and Noks1 *Ha*CR1 share a 96.9% amino acid sequence identity and 98.4% sequence similarity, but *Ha*CR2 comprises an additional 89-amino-acid insertion in the middle part of the protein (Cabral *et al.*, 2011). Consistent with the absence of *HaCR2* for the Noks1 genome sequence, we could not amplify a *HaCR2* orthologue by RT-PCR. We therefore concluded that there is no *HaCR2* orthologue existing in the strain Noco2. *Ha*CR1 and its closest homologue *Ha*CR3 (BLASTp E-value 9×10^–30^) share 53.4% sequence identity and 61.1% sequence similarity (Supplementary Fig. S9B) on the amino acid level. *Ha*CR1 is unique to the species of *H. arabidopsidis*, because we did not find any *Ha*CR1 homologue in another oomycete species by BLASTp search against the NCBI database (E-value cut-off ≤1). As *HaCR3* shared also 68.2% of transcript sequence identity to *HaCR1* (Supplementary Fig. S10A), we sought to examine co-suppression of *HaCR3* in *HaCR1*^*RNAi*^ plants upon *H. arabidopsidis* infection. We performed qRT-PCR for gene expression analysis and observed comparable *HaCR3* transcript accumulation in WT and *HaCR1*^*RNAi*^ plants at 4 dpi (Supplementary Fig. S10B), suggesting that *HaCR1*^*RNAi*^ was specific to knockdown *HaCR1*, but not *HaCR3*.

### *Ha*CR1 inhibits induced plant cell death and promotes infection by (hemi)biotrophs

**Fig. 2.**
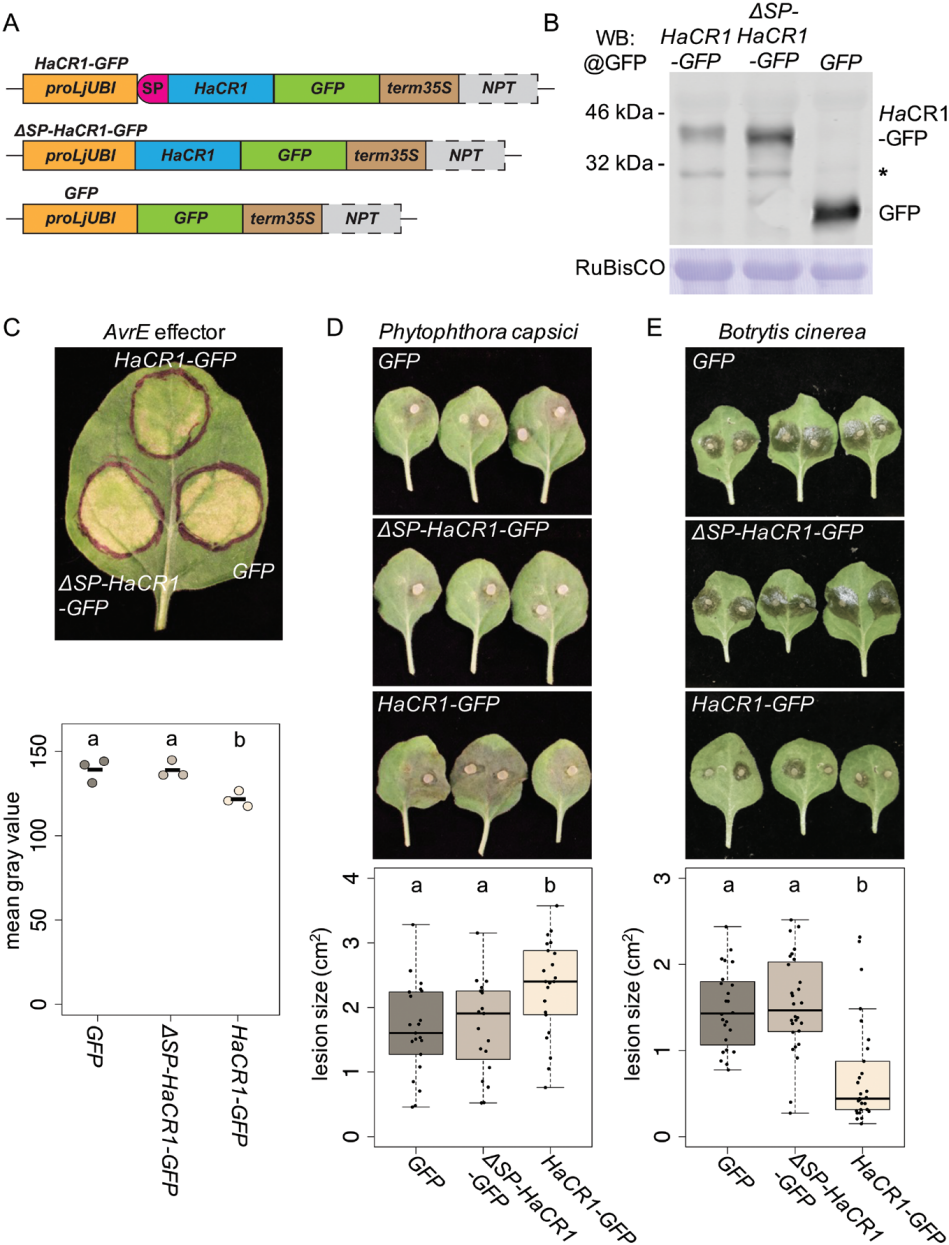
Expression of full-length *Ha*CR1 in *N. benthamiana* suppresses effector-triggered plant cell death and promoted disease of *P. capsici* but reduced disease of *B. cinerea*. (A) Schematic overview of *Ha*CR1 expression cassettes: C-terminal *GFP* fused to full-length *HaCR1*, C-terminal *GFP* fused to a *HaCR1* version without signal peptide (⊿*SP*), and *GFP* without *HaCR1*. *ProLjUBI* is a *Lotus Ubiquitin* promoter, *SP* represents signal peptide, *term35S* is a S35 viral terminator, *NPT* is a *Neomycin-phosphotransferase* resistance gene (only included when transforming Arabidopsis). (B) Western blot analysis confirmed expression of *Ha*CR1–GFP, ∆SP–*Ha*CR1–GFP fusion proteins or GFP in *A. tumefaciens*-infiltrated tobacco leaves. The expected size of *Ha*CR1–GFP was 40.6 kDa, of ∆SP–*Ha*CR1–GFP was 38.7 kDa, and of free GFP was 26.9 kDa. Asterisk indicates a non-specific band. RuBisCO stained with Coomassie G250 was used as a loading control. (C) A representative picture of a tobacco leaf at 5 d after *A. tumefaciens* co-infiltration carrying either *HaCR1-GFP*, *∆SP-HaCR1-GFP* or *GFP* together with a construct carrying the bacterial effector *AvrE* promoting cell death. This experiment was repeated three times with comparable results. Each experiment included three infiltrated leaves. Quantification of chlorosis symptoms was performed by measuring the mean grey value of the infiltrated area, with *n*=3. (D) *Agrobacterium tumefaciens*-infiltrated *N. bethamiana* leaves of *HaCR1-GFP*, ⊿*SP-HaCR1-GFP*, or *GFP* were inoculated with *P. capsici*, and pictures were taken at 2 dpi. Lesion size quantification on *N. benthamiana* leaves induced by *P. capsici* at 2 dpi, as determined by ImageJ with *n*≥20 lesions of *n*≥10 leaves. (E) *Agrobacterium tumefaciens*-infiltrated *N. benthamiana* leaves of *HaCR1-GFP*, ⊿*SP-HaCR1-GFP*, or *GFP* were inoculated with *B. cinerea*, and pictures were taken at 3 dpi. Lesion size quantification on *N. benthamiana* leaves induced by *B. cinerea* at 3 dpi, as determined by ImageJ with *n*≥20 lesions of *n*≥10 leaves. Letters in (C–E) indicate groups of statistically significant difference by ANOVA followed by Tukey’s HSD test with *P*≤0.05.

**Fig. 3.**
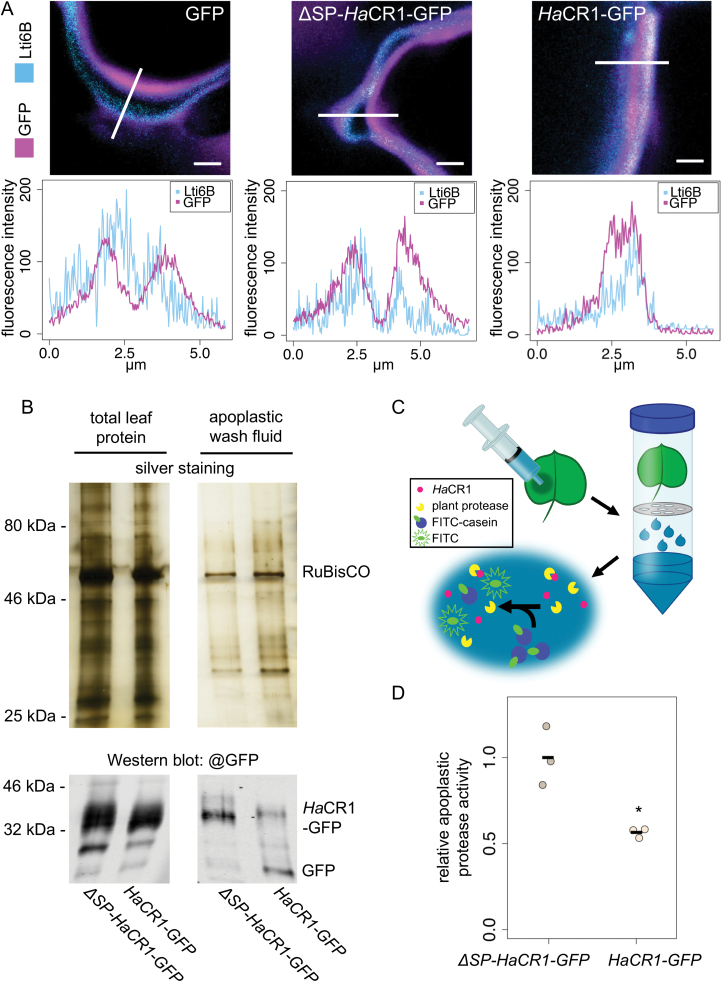
*Ha*CR1 localizes to the plant apoplast and is capable of inhibiting apoplastic plant protease activity. (A) Confocal laser scanning microscopy was used to inspect intercellular localization of *Ha*CR1–GFP, ∆SP–*Ha*CR1–GFP, or GFP alone. The plasma membrane (PM) was visualized by the PM marker LTi6B fused with CFP. Six independent events of co-localization were evaluated per construct. Scale bars represent 100 µm. The upper panel displays overlaid LTi6B–CFP and *Ha*CR1–GFP, ∆SP–*Ha*CR1-GFP, or GFP fluorescence signal intensities alongside the bar, as indicated in the fluorescence microscopy images (lower panel). (B) Total leaf protein and apoplastic wash fluid fraction visualized via a silver-stained SDS-PAGE indicated depletion of cytoplasmic proteins, such as RuBisCO, from the apoplastic wash fluid. Western blot shows detection of *Ha*CR1 and free GFP in total leaf and apoplastic wash fluid from the same experiment. (C) Schematic overview of the apoplastic protease activity assay. *Nicotiana benthamiana* leaves were transformed by *A. tumefaciens* infiltration, apoplastic wash fluid was collected by centrifugation, and endogenous protease activity was determined by addition of FITC-casein. Upon protease activity, casein is hydrolysed and quenching of FITC fluorescence is released. (D) Protease activity in the apoplastic wash fluid was measured and compared with apoplastic proteins collected from *N. benthamiana* leaves expressing *Ha*CR1–GFP or ∆SP–*Ha*CR1–GFP. Protease activity was normalized to total protein quantities of apoplastic fluid samples. Each data point represents an independent experiment using eight leaves. *P≤0.05, significant difference by Student’s *t*-test.

In order to shed light on *Ha*CR1 function during plant infection, we performed transient expression assays using *N. benthamiana* leaves. We cloned a full-length *HaCR1* version and fused it with a C-terminal *GFP* tag (*HaCR1-GFP*), a C-terminal *GFP*-tagged *HaCR1* version without its predicted signal peptide (*∆SP-HaCR1-GFP*), and *GFP* without any *Ha*CR1 sequence as a negative control (*GFP*) for expression in *N. benthamiana* leaves (Fig. 2A, B). Because *HaCR1* knockdown by HIGS resulted in plant trailing necrosis, we hypothesized that *Ha*CR1 might promote infection through supressing plant cell death. To test this hypothesis, we co-expressed the *HaCR1-GFP* or the *∆SP-HaCR1-GFP* construct together with the *P. syringae* effector *AvrE*, a known trigger of plant cell death in *N. benthamiana* (Badel *et al.*, 2006). Only *HaCR1-GFP* was able to dampen *AvrE1*-induced plant cell death in contrast to both *∆SP-HaCR1-GFP* and *GFP* (Fig. 2C). To further substantiate the role of *Ha*CR1 as a plant cell death inhibitor for plant infection, we inoculated *HaCR1-GFP*-infiltrated *N. benthamiana* leaves with the hemibiotrophic oomycete pathogen *P. capsici* or with the necrotrophic fungal pathogen *B. cinerea.* These two pathogens lack any homologous protein with sequence similarity to *Ha*CR1 (no BLASTp hit with E-value ≤5). *Phytophthora capsici* generated significantly larger lesions in *HaCR1-GFP* expressing leaves, compared with *∆SP-HaCR1-GFP* or *GFP* expressing leaves (Fig. 2D). In contrast, *B. cinerea*, the infection of which is supported by induced plant cell death (Govrin and Levine, 2000), produced significantly smaller lesions in *HaCR1-GFP* expressing leaves than in *∆SP-HaCR1-GFP* or in *GFP* expressing leaves (Fig. 2E). Since *Ha*CR1 does not contain any RxLR plant cell translocation motif, we postulated that it could be active in the plant apoplast. To inspect *Ha*CR1 intercellular localization in plants, we co-expressed a CFP-fused protein of the known plant plasma membrane marker *Lti6B* (Kurup *et al.*, 2005) with *HaCR1-GFP*, *∆SP-HaCR1-GFP*, or *GFP* in *N. benthamiana* leaves. Confocal microscopy studies revealed overlapping GFP and CFP signals for *Ha*CR1–GFP indicating co-localization with Lti6B and the presence of *Ha*CR1 in both the plant apoplast and the symplast, while ∆SP*–Ha*CR1*–*GFP or GFP indicated signals separate from Lti6B and seemingly located only in the plant symplast (Fig. 3A). These results demonstrated that *Ha*CR1 was functional in suppressing induced plant cell death, and its signal peptide was crucial for this function.

### *Ha*CR1 might act as an apoplastic protease inhibitor to support infection

A previously described role of fungal CR proteins is the inhibition of apoplastic plant protease (Rooney *et al.*, 2005). Therefore, we hypothesized that *Ha*CR1 might function as a decoy to inhibit plant apoplastic proteases, too. To challenge this hypothesis, we measured the capacity of *Ha*CR1 to interfere with the apoplastic plant protease activity *in vitro*. We collected apoplastic wash fluids from *N. benthamiana* leaves expressing either *HaCR1-GFP* or *∆SP-HaCR1-GFP*. Comparative analysis of the total leaf versus the apoplastic proteins by SDS-PAGE and silver staining displayed a reduction of the intracellular protein ribulose-1,5-bisphosphate carboxylase/oxygenase (RuBisCO) in the apoplast fraction indicating successful enrichment of apoplastic proteins, even though we could not entirely prevent cytoplasmic protein contamination, as RuBisCO and *∆SP-HaCR1-GFP* were still detectable in apoplast samples (Fig. 3B). Indeed, the apoplastic wash collected from *N. benthamiana* leaves expressing *HaCR1-GFP* exhibited a significant reduction of plant protease activity determined by fluorescein isothiocyanate (FITC)–casein compared with *∆SP-HaCR1-GFP* (Fig. 3C, D). This result further supported a function of *Ha*CR1 in the plant apoplast.

**Fig. 4.**
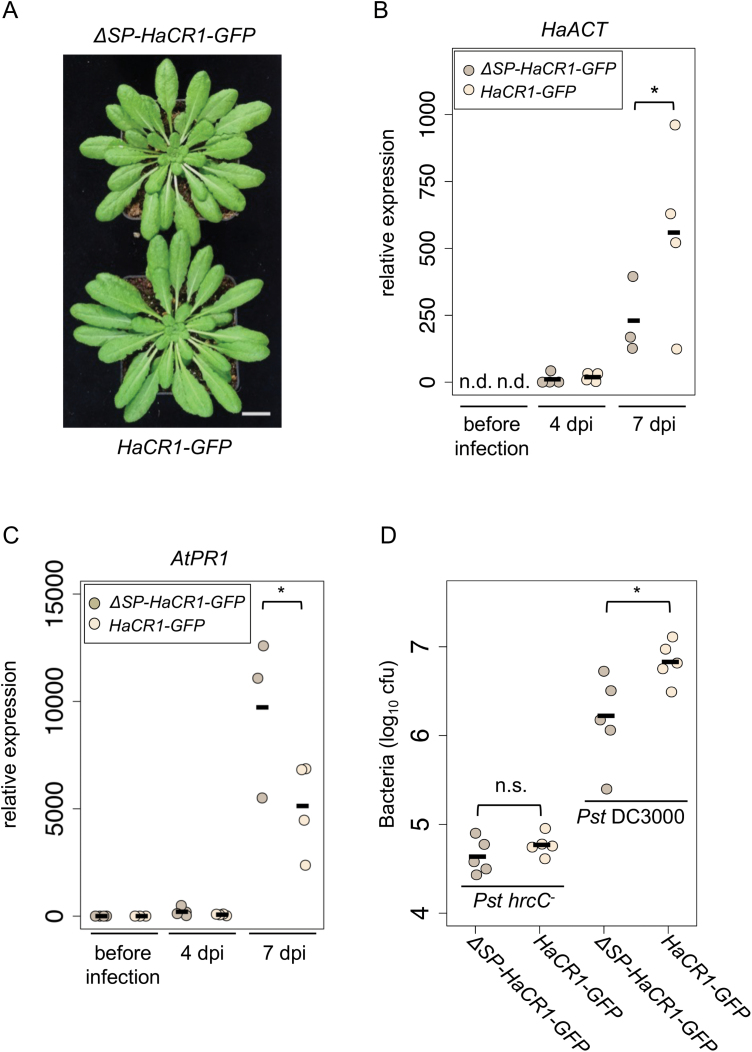
Ectopic expression of *Ha*CR1 in Arabidopsis enhances susceptibility to *H. arabidopsidis* and bacterial infection and compromises plant immunity. (A) Growth of Arabidopsis plants overexpressing either *∆SP-HaCR1-GFP* or *HaCR1-GFP*. The scale bar represents 2 cm. (B) *H. arabidopsidis* biomass was determined by *HaACT* expression relative to plant *AtACT* in *HaCR1-GFP* expressing Arabidopsis, compared with *∆SP-HaCR1-GFP* at 4 and 7 dpi. *HaACT* expression was not detected (n.d.) before infection. (C) *AtPR1* was quantified by qRT-PCR using *AtACT2* and *AtTUB* as reference genes, and relative transcript levels were compared between Arabidopsis expressing *HaCR1-GFP* or *∆SP-HaCR1-GFP* at 4 and 7 dpi with *H. arabidopsidis*. (D) Arabidopsis susceptibility to the virulent *Pst* DC3000 or the avirulent *Pst* DC3000 *hrcC*^*−*^ was evaluated by counting colony forming units (cfu) at 3 dpi. Each data point represents cfu derived from three infected leaf discs. For (B, C), each experiment was performed at least with three biological replicates, and each biological replicate represented two technical repeats. For (B–D) *P≤0.05, significant difference by one-tailed Student’s *t*-test.

To investigate the suppressive effect of *Ha*CR1 on plant immunity in the native host Arabidopsis during *H. arabidopsidis* infection, we generated transgenic Arabidopsis plants expressing *HaCR1-GFP* or *∆SP-HaCR1-GFP* under the strong constitutive *Lotus Ubiquitin* promoter (Maekawa *et al.*, 2008). We recovered three independent Arabidopsis T_2_ lines for *HaCR1-GFP* and two independent lines for *∆SP-HaCR1-GFP* and verified ectopic expression of fusion proteins in seedlings by fluorescence microscopy and western blot analysis (see Supplementary Fig. S11A, B). None of the transgenic lines exhibited any obvious growth or morphological change (Fig. 4A). We pooled and germinated seeds of the corresponding transgenic lines and challenged seedlings with the virulent *H. arabidopsidis* Noco2. Disease progression was estimated by *H. arabidopsidis* housekeeping gene expression of *HaACT A* relative to plant *AtACT 2* at 4 and 7 dpi. Moderate but significantly increased pathogen quantity was evident in *HaCR1-GFP* expressing seedlings, compared with *∆SP-HaCR1-GFP* (Fig. 4B). Moreover, expression of the Arabidopsis salicylic acid (SA)-dependent immunity marker gene *AtPR1* was significantly less induced in seedlings expressing *HaCR1-GFP* compared with *∆SP-HaCR1-GFP* upon *H. arabidopsidis* infection (Fig. 4C). This finding supported a role of the full-length *Ha*CR1 in plant immune suppression. The jasmonic acid-dependent immunity marker gene *AtPDF1.2* did not exhibit any difference between *HaCR1-GFP* and *∆SP-HaCR1-GFP* upon *H. arabidopsidis* infection (Supplementary Fig. S12). To further explore if the *Ha*CR1-suppressive effect on SA-dependent immunity played a role during infection, we inoculated transgenic Arabidopsis lines either with the virulent bacterial hemibiotrophic pathogen *Pseudomonas syringae* pv. *tomato* (*Pst*) strain DC3000 or the avirulent mutant *Pst* DC3000 *hrcC*^*−*^ lacking a functional type-III secretion system (Roine *et al.*, 1997). Bacterial growth of DC3000 was significantly enhanced in *HaCR1-GFP* expressing Arabidopsis, compared with *∆SP-HaCR1-GFP*. In contrast, bacterial population of *hrcC*^*−*^ remained unaltered between the two different transgenic plant lines (Fig. 4D). These results further supported that *Ha*CR1 is an apoplastic effector that impairs plant immunity against diverse biotrophic and hemibiotrophic plant pathogens.

## Discussion

In this study, we used Arabidopsis HIGS for functional gene studies in the obligate biotrophic pathogen *H. arabidopsidis*. The short lifecycle, available cloning tools, and easy transformation of the host plant Arabidopsis enables the conducting of HIGS experiments in a relatively short time period. We applied HIGS to knockdown *HaACT A*, *HaDCL1*, *HaCR1*, and *HaA1E* in order to survey functional roles of these pathogen genes during host plant colonization. The *HaCR1*^*RNAi*^, *HaACT*^*RNAi*^ plants, and to a lesser extent *HaA1E*^*RNAi*^, exhibited trailing necrosis at *H. arabidopsidis* infection sites. In addition, *HaCR1*^*RNAi*^ and *HaACT*^*RNAi*^ plants allowed reduced proliferation of oospores (Fig. 1A–C; Supplementary Fig. S4A), the sexual reproductive structure of oomycetes (Slusarenko and Schlaich, 2003). Both infection phenotypes are related to reduced disease, as comparable trailing necrosis symptoms had been observed when Arabidopsis was primed for immunity, or connected to Arabidopsis ecotypes infected with sub-compatible *H. arabidopsidis* strains (Uknes *et al.*, 1992; Krasileva *et al.*, 2011). The resistance response in *HaACT*^*RNAi*^ plants also suggested that down-regulation of *HaACT A* was not compensated through functional redundancy by the paralogue *HaACT B* (*HpaG809873*), despite considerable sequence homology with the *HaACT*^*RNAi*^ HIGS construct (see Supplementary Fig. S13A). The attenuated disease development in *HaACT*^*RNAi*^, *HaCR1*^*RNAi*^, and *HaA1E*^*RNAi*^ plants was not due to plant transformation or due to expression of non-self dsRNA in Arabidopsis, because *HaDCL1*^*RNAi*^ did not reveal any higher plant resistance or suppressed pathogen virulence (Supplementary Fig. S4B). Why *HaDCL1*^*RNAi*^ plants did not reveal higher resistance despite the important role of pathogen small RNAs during infection (Dunker *et al.*, 2020) remains to be investigated. One possible explanation might be functional redundancy of the two *Ha*DCLs identified in the genome of *H. arabidopsidis* (Bollmann *et al.*, 2016). Similarly, the mild phenotype expressed in the Arabidopsis *HaA1E*^*RNAi*^ line might be explained by the presence of two paralogous and potentially functionally redundant genes: *HaA1E-LIKE* (*HaA1EL*, *HpaG807738*) and *HaA1E-LIKE2* (*HaA1EL2*, *HpaG807727*). *Ha*A1EL shares 66.3% amino acid identity and 79.6% amino acid similarity, while *Ha*A1EL2 shares 86.6% amino acid identity and 90.3% amino acid similarity with *Ha*A1E. *HaA1EL* and *HaA1EL2* showed also 79.4% and 89.9% coding sequence identity, respectively. In addition, they revealed a considerable DNA sequence homology with our *HaA1E*^*RNAi*^ HIGS construct (Supplementary Fig. S13B). However, *Ha*A1EL2 lacks an annotated open reading frame with a signal peptide and displayed very weak expression during infection. In contrast *Ha*A1E and *Ha*A1EL both comprise a predicted signal peptide and were previously found to be strongly expressed in *H. arabidopsidis* infecting Arabidopsis (Asai *et al.*, 2014).

At the molecular level, target gene suppression of *HaACT* and *HaCR1* by HIGS was evident at 4 dpi, but not at 7 dpi (Fig. 1D; Supplementary Fig. S7). At this later time point, *H. arabidopsidis* had induced trailing necrosis of plant cells at the infection sites of HIGS plants. We suggest that plant cell death would lead to a collapsed haustoria–plant cell interface, which likely stopped the transport of RNAs from plants into the pathogen (Hudzik *et al.*, 2020). Transgenic *HaACT*^*RNAi*^ expressing Arabidopsis displayed pleiotropic effects, for instance slower plant growth, although we did not predict any Arabidopsis *ACTIN* as off-target of the *HaACT*^*RNAi*^ construct *in silico*, and the two closest orthologues of *HpaACT A*, *AtACT2* and *AtACT11*, were not suppressed in the Arabidopsis HIGS line (Supplementary Fig. S8). Tracing back off-target effects would require plant RNA degradome analysis (Casacuberta *et al.*, 2015), and was not further investigated as it would go beyond the scope of our study. With this experience, we propose to omit pathogen house-keeping genes as targets in HIGS studies although successful silencing would likely promote plant disease resistance. Since pathogen effector genes are unique and homologues do not exist in the host plant, HIGS against *HaCR1* did not encounter any off-target problem. An interesting alternative to HIGS for targeted gene knock-down in *H. arabidopsidis* that is based on exogenous application of 5′ capped small interfering RNAs has been recently reported (Bilir *et al.*, 2019). Applying *Cellulose synthase A3* antisense RNAs to *H. arabidopsidis* conidia suspension inhibited spore germination on the leaf surface. Both, transgenic HIGS and external RNA treatment are innovative strategies to further explore gene functions of this pathogen.

To further investigate the role of *Ha*CR1 during plant infection, we expressed *Ha*CR1 in *N. benthamiana* and Arabidopsis. One obvious disease symptom in transgenic *HaCR1*^*RNAi*^ plants was the induction of local plant cell death suggesting that *Ha*CR1 might be involved in cell death suppression. Such a function of *Ha*CR1 was supported by the inhibitory activity on bacterial effector AvrE-induced plant cell death in *N. benthamiana* leaves (Fig. 2C). Of note, full-length *Ha*CR1, but not a signal peptide-deleted version, was capable of suppressing plant cell death in this assay. AvrE expressed in *N. benthamiana* leaves was previously described to be localized at the cell plasma membrane (Xin *et al*., 2015), a possible contact compartment of *in planta*-expressed full length *Ha*CR1 with AvrE. However, the molecular mechanism of AvrE-induced cell death repression by *Ha*CR1 is not clear, and needs to be further explored by identifying the molecular interactors of *Ha*CR1. On the one hand, *Ha*CR1 promoted disease caused by the oomycete hemibiotrophic pathogen *P. capsici* (Fig. 2D). We suggest that *P. capsici* profits from *Ha*CR1-repressed plant cell death during the early biotrophic phase. This is in line with Avr1b from *Phytophthora sojae* that impaired plant cell death and promoted lesion formation of this hemibiotrophic pathogen (Dou *et al.*, 2008). On the other hand, *Ha*CR1 expression limited disease symptoms caused by the necrotrophic fungal pathogen *B. cinerea* (Fig. 2E), because this pathogen exploits and promotes plant apoptosis for infection (Veloso and van Kan, 2018). In this context, reduced *Botrytis* virulence was reported in plants expressing animal cell death suppressors (Dickman *et al.*, 2001). Consistent with plant cell death suppressive activity, *Ha*CR1 also promoted disease progression caused by other (hemi)biotrophic pathogens, *P. syringae* DC3000 and *H. arabidopsidis* itself (Fig. 4B, E). *Ha*CR1 overexpression in Arabidopsis moderately promoted *H. arabidopsidis* disease, which might be explained by the high expression of endogenous *Ha*CR1 in *H. arabidopsidis* during infection. Similarly, a previous study on *Ha*RxLR effectors could detect only small positive effects on *H. arabidopsidis* infection suggesting a combined action of effectors to effectively suppress plant immunity (Pel *et al.*, 2014).

To better understand the molecular function of *Ha*CR1, we explored its peptide composition. *Ha*CR1 contains a predicted secretion signal peptide but no further plant cell translocation domain indicating its function in the plant apoplast. In accordance, only full-length *Ha*CR1 expression in plants suppressed induced plant cell death and promoted infection of (hemi)biotrophs, while a secretion signal peptide-truncated *Ha*CR1 version expressed in plants lost these activities. This is in agreement with other apoplastic effectors found in fungal pathogens, such as *Zymoseptoria tritici* and *Magnaporthe oryzae* (Kim *et al.*, 2013; Kettles *et al.*, 2017). A conserved class of apoplastic effectors in fungi are the LysMs. These effectors act as decoys that prevent microbe-associated molecular pattern (MAMP)-triggered plant immunity, for instance by binding chitin oligomers and thereby hampering chitin recognition by plant pattern receptors (Kombrink and Thomma, 2013; Zeng *et al.*, 2020). However, *Ha*CR1, like all other members of the *Ha*CR family, does not contain any predicted protein domain or motif, making a specific ligand binding rather unlikely. Instead, we found evidence that plant-expressed *Ha*CR1 can interfere with apoplastic plant protease activity *in vitro* (Fig. 3D), similar to fungal CR proteins exhibiting protease inhibition activity (Rooney *et al.*, 2005). The strict dependency of *Ha*CR1 function on the presence of a signal peptide, and thereby its apoplastic localization, suggests a link between the cell death inhibition function and plant protease inhibition. In this context, several apoplastic plant proteases are crucial regulatory components of plant programmed cell death, and protease inhibitory effectors of fungi and oomycetes have been associated with inhibition of plant programmed cell death (Dickman and Fluhr, 2013; Salguero-Linares and Coll, 2019).

In general, small, apoplastic CR peptides containing no further sequence-conserved domains have been described in high numbers for oomycete and fungal pathogens suggesting a functional conservation in a wide range of pathogens (Sperschneider *et al.*, 2018). Pathogen-secreted protease inhibitors or decoys prevent degradation of pathogen effectors or the release of MAMPs produced by plant proteases (Jiang and Tyler, 2012). Such a function of *Ha*CR1 is supported by our results, because Arabidopsis overexpressing *Ha*CR1 exhibited reduced *AtPR1* induction upon *H. arabidopsidis* infection (Fig. 4C), and *Ha*CR1 expression in plants promoted infection of (hemi)biotrophic pathogens *H. arabidopsidis*, *P. capsici*, and *P. syringae*.

Our data revealed an important role of a CR effector protein in host infection by the obligate biotrophic pathogen *H. arabidopsidis*. A next crucial step to understand the molecular mechanism of how *Ha*CR1 suppresses pathogen-induced plant cell death will be to uncover its molecular interactors, which are likely to include plant apoplastic proteases or receptor-like proteins. This knowledge would be crucial to completely elucidate whether the dual function of *Ha*CR1 in plant protease inhibition and cell death inhibition is directly or indirectly linked, or is independent.

## Supplementary data

Supplementary data are available at *JXB* online.

Fig. S1. Expression correlation analysis of *H. arabidopsidis* and Arabidopsis reference genes by qRT-PCR.

Fig. S2. Expression of *GFP*^*RNAi*^ by *A. tumefaciens* infiltration led to *GFP* silencing in the *N. benthamiana* line 16c stably expressing *GFP*.

Fig. S3. Arabidopsis *HaACT*^*RNAi*^ and *HaCR1*^*RNAi*^ plants displayed no obviously altered infection phenotype at 4 dpi.

Fig. S4. Infection phenotype of the Arabidopsis *HaA1E*^*RNAi*^ and the *HaDCL1*^*RNAi*^ lines.

Fig. S5. Representative leaves used for *H. arabidopsidis* oospore quantification.

Fig. S6. Validation of RT-PCR primers to assess *H. arabidopsidis* target gene expression in Arabidopsis HIGS plants.

Fig. S7. *H. arabidopsidis* target gene expression when infecting Arabidopsis HIGS plants.

Fig. S8. Growth phenotype of 14-day-old Arabidopsis WT, *HaCR1*^*RNAi*^ or *HaACT*^*RNAi*^ seedlings.

Fig. S9. The *Ha*CR family in the *H. arabidopsidis* strain Noco2.

Fig. S10. *HaCR3* expression was not suppressed during infection of *HaCR1*^*RNAi*^ plants.

Fig. S11. Arabidopsis seedlings of individual transformation lines expressing *HaCR1-GFP* or *∆SP-HaCR1-GFP*.

Fig. S12. Expression of *AtPDF1.2* was not different when comparing *∆SP-HaCR1-GFP* or *HaCR1-GFP* expressing Arabidopsis seedlings upon *H. arabidopsidis* infection.

Fig. S13. Sequence alignment of RNAi constructs with the target gene and the closest homologues.

Table S1. Primers used in this study.

eraa472_suppl_Supplementary_Figures_S1-S13

eraa472_suppl_Supplementary_Tables

## Data Availability

All data supporting the findings of this study are available within the paper and within its Supplementary data published online. All plasmids and transgenic plant lines created during this study are available from the corresponding author (AW) upon reasonable request.

## Abbreviations

CFP: cyan fluorescent protein
CR: cysteine-rich
dpi: days post-inoculation
FITC: fluorescein isothiocyanate
GFP: green fluorescent protein
HIGS: host-induced gene silencing
NLR: nucleotide-binding oligomerization domain-like receptor
*R* gene: resistance gene
RNAi: RNA interference
qRT-PCR: quantitative reverse transcription–polymerase chain reaction
Pst: *Pseudomonas syringae* pv *tomato*
SA: salicylic acid
SP: signal peptide
WT: wild type
YFP: yellow fluorescent protein
