# Supplementary material for: An Arabidopsis downy mildew non-RxLR effector suppresses induced plant cell death to promote biotroph infection
Source: J Exp Bot. 2020 Oct 16;72(2):718–32. doi: 10.1093/jxb/eraa472 (PMC7853606; doi:10.1093/jxb/eraa472)
Supplement: eraa472_suppl_Supplementary_Figures_S1-S13 [file eraa472_suppl_supplementary_figures_s1-s13.pdf]

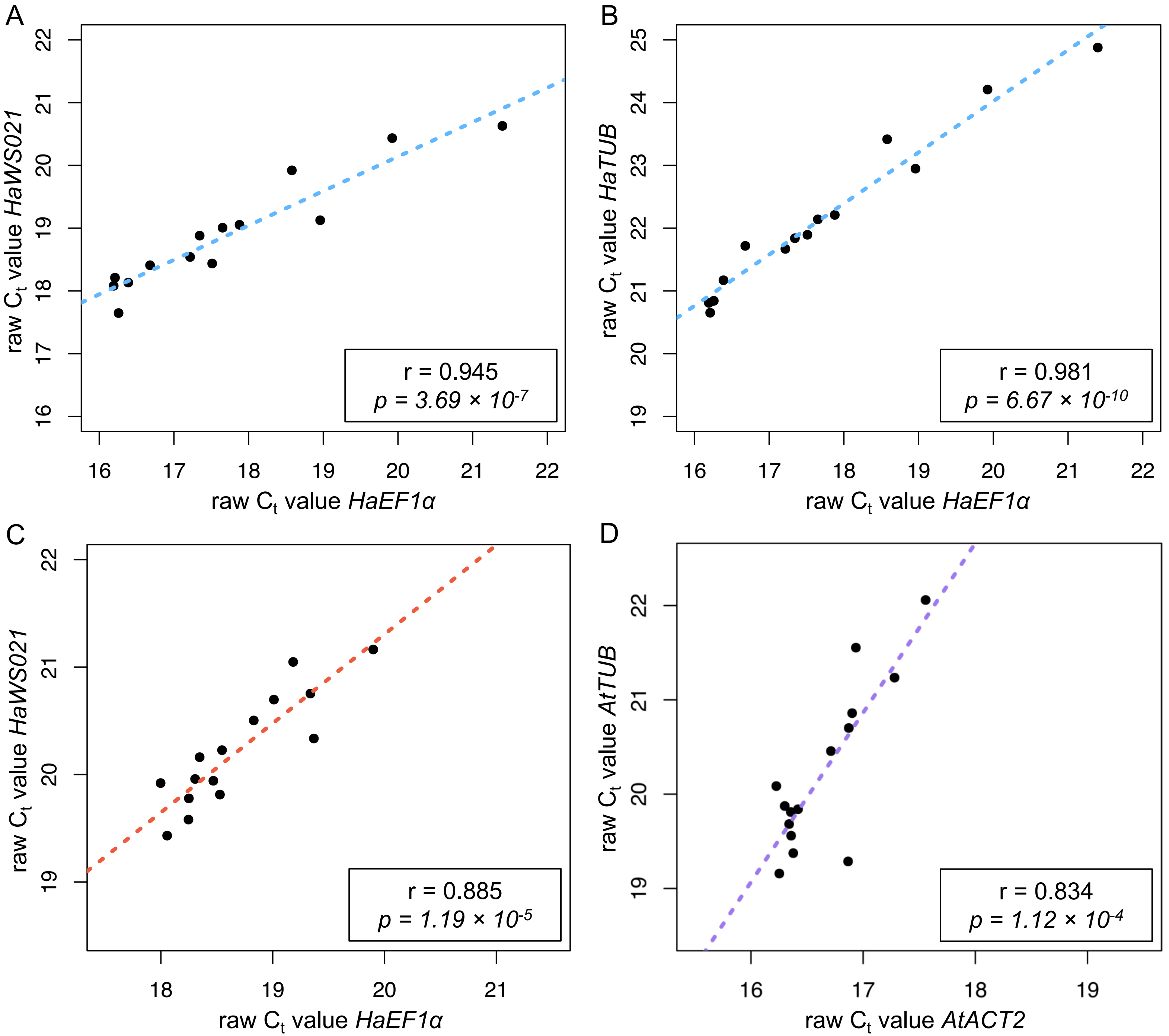

Figure S1: Expression correlation analysis of *H. arabidopsidis* and *A. thaliana* reference genes by qRT-PCR. Raw C_t values of three *H. arabidopsidis* reference genes and two *A. thaliana* reference genes were recorded in infected *A. thaliana plants*. A) Correlation plot of C_t values between A) *HaEF1α* and *HaWS021,* B) *HaEF1α* and *HaTUB* and C) *HaEF1α* and *HaWS021* are displayed. C_t values in A) and B) were measured at 4 dpi and in C) at 7 dpi. Despite considerable variation in the C_t values between samples reflecting the variation of the *H. arabidopsidis* infection strength, expression of the three genes was highly correlated, validating their stable expression. D) Ct values of the two *A. thaliana* reference genes *AtACT2* and *AtTUB* displayed correlated expression. Data were recorded from expression resembling pooled samples at 4 and 7 dpi. The boxes in A) - D) display the Pearson's correlation coefficient r and p-value of correlation for each correlation analysis.

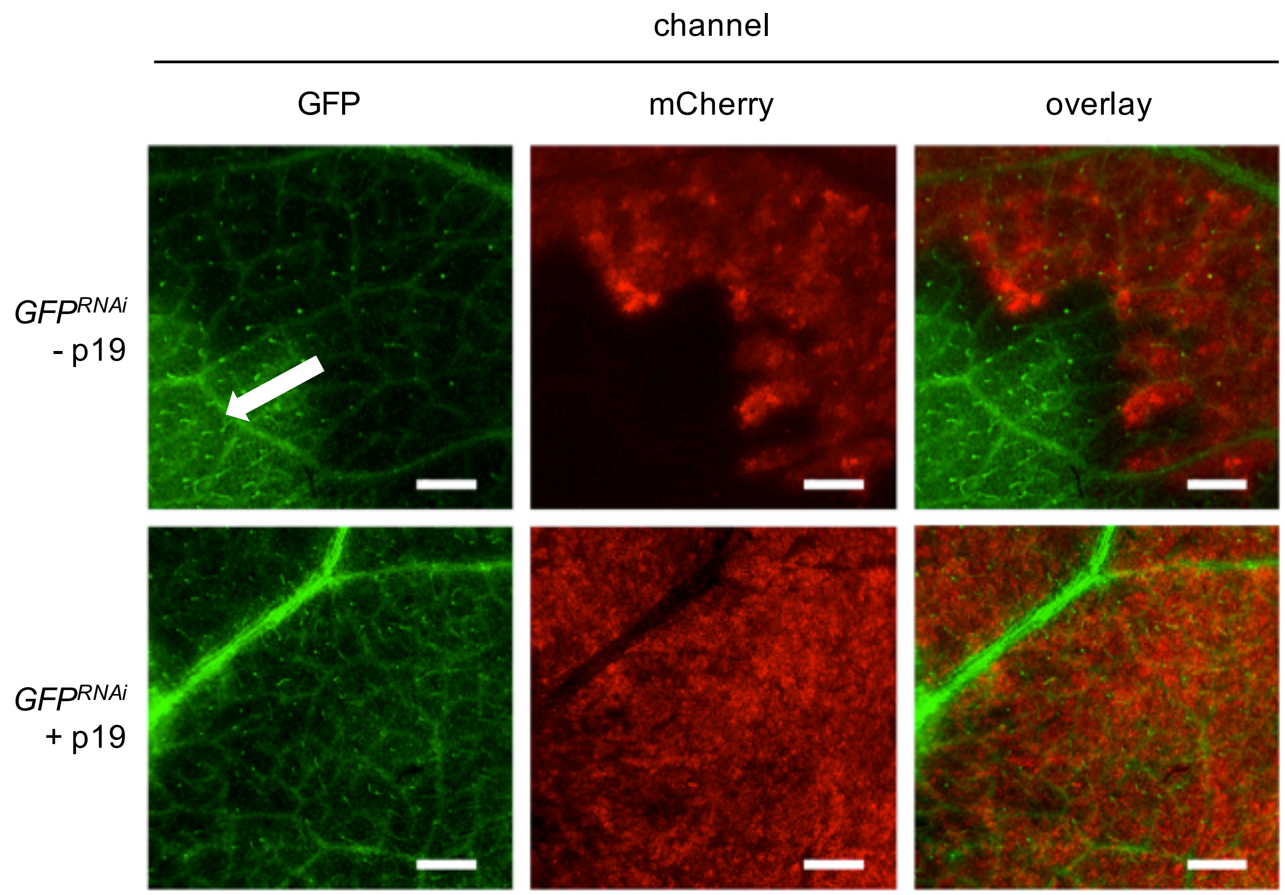

Figure S2: Expression of *GFP*^{RNAi} by *A. tumefaciens* infiltration led to *GFP* silencing in the *N. benthamiana* line 16c stably expressing *GFP*. Epifluorescence pictures were taken 2 days post infiltration, mCherry was used as a transformation marker. The arrow displays the endogenous GFP fluorescence of line 16c in the untransformed region. Co-expression of the viral RNAi suppressor p19 was used to validate RNAi-dependent gene silencing. The scale bars indicates 1 mm.

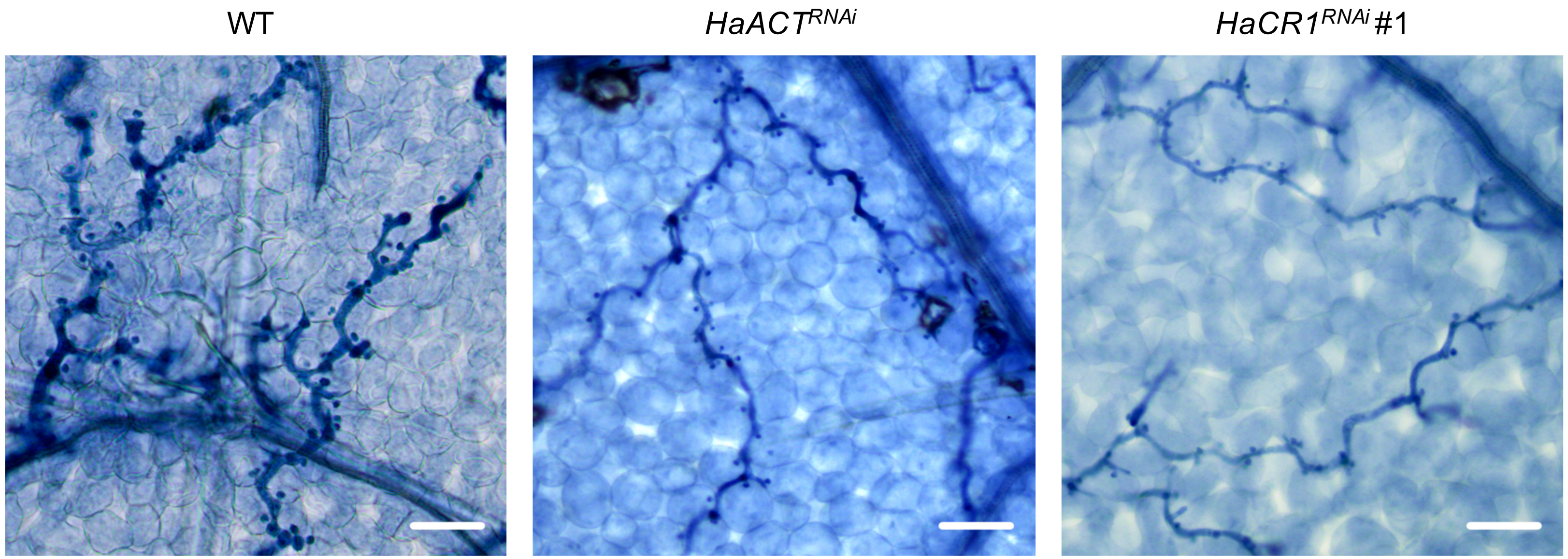

Figure S3: *A. thaliana HaACT*^{RNAi} and *HaCR1*^{RNAi} plants displayed no obviously altered infection phenotype at 4 dpi. A minimum of five leaves was inspected per genotype. Scale bars represent 50 µm.

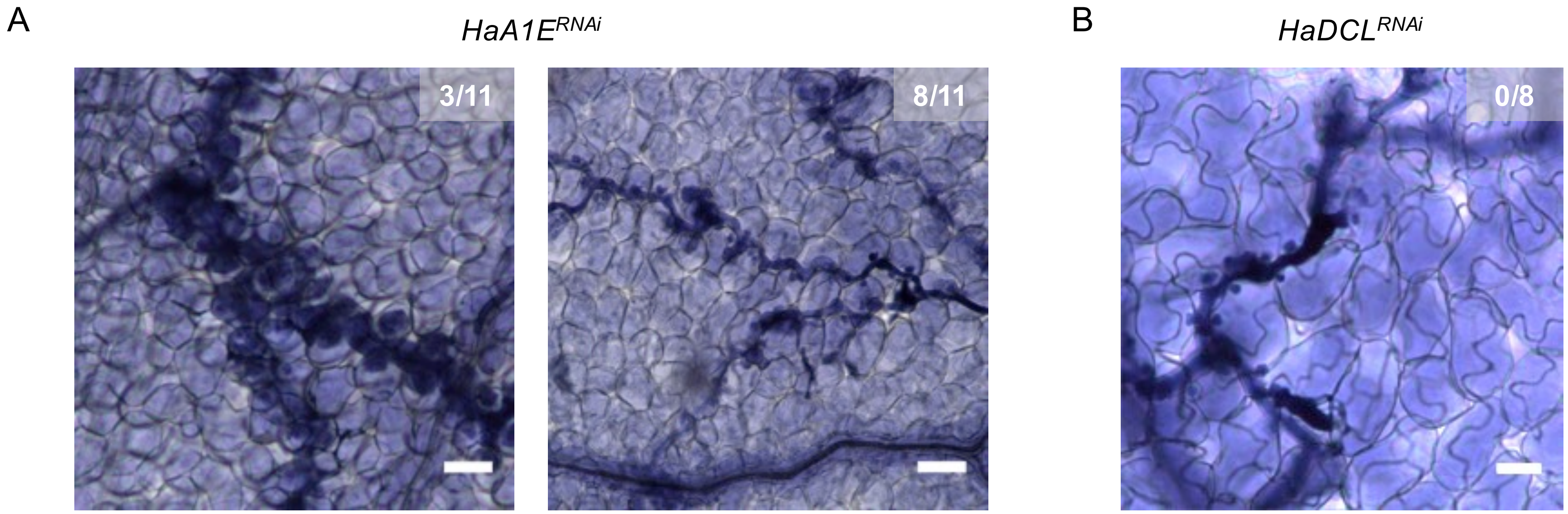

Figure S4: Infection phenotype of the *A. thaliana HaA1E^{RNAi}* and the *HaDCL1^{RNAi}* lines. A) The *A1E^{RNAi}* line exhibited moderate resistance against *H. arabidopsidis*. At 7 dpi, trailing necrosis was detected in three out of eleven infected seedling leaves, while eight out of eleven leaves displayed no trailing necrosis. B) *HaDCL1^{RNAi}* plants did not display trailing necrosis upon infection with *H. arabidopsidis*. Scale bars represent 50 µm.

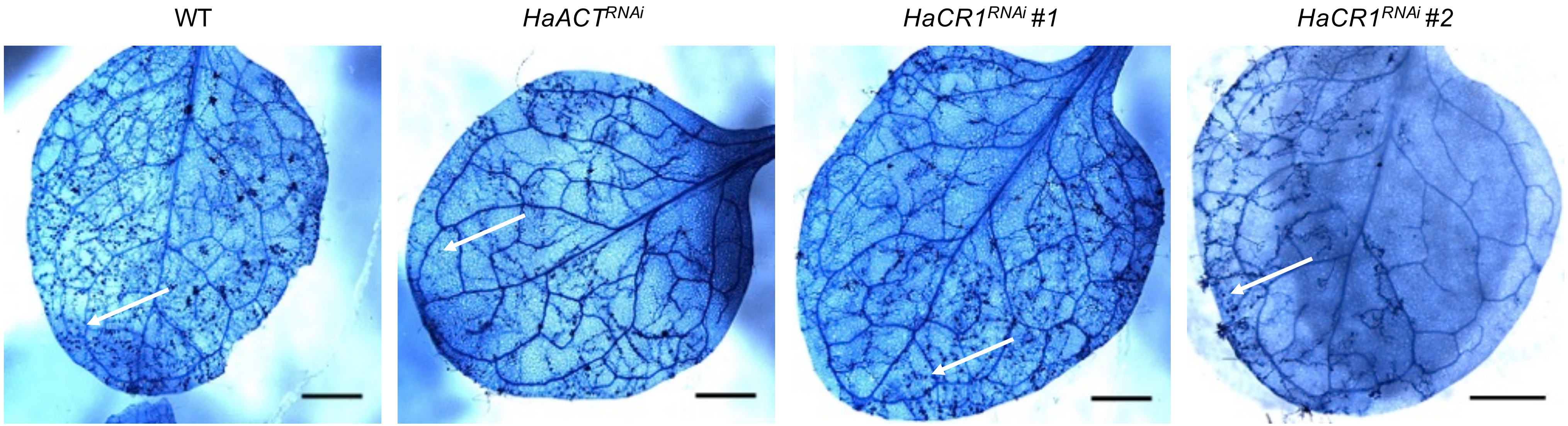

Figure S5: Representative leaves used for *H. arabidopsidis* oospore quantification. *A. thaliana HaACT*^{RNAi} and both *HaCR1*^{RNAi} lines allowed reduced oospore production. The white arrow indicates an oospore. Leaves were inspected at 7 dpi. Scale bars indicate 2 mm.

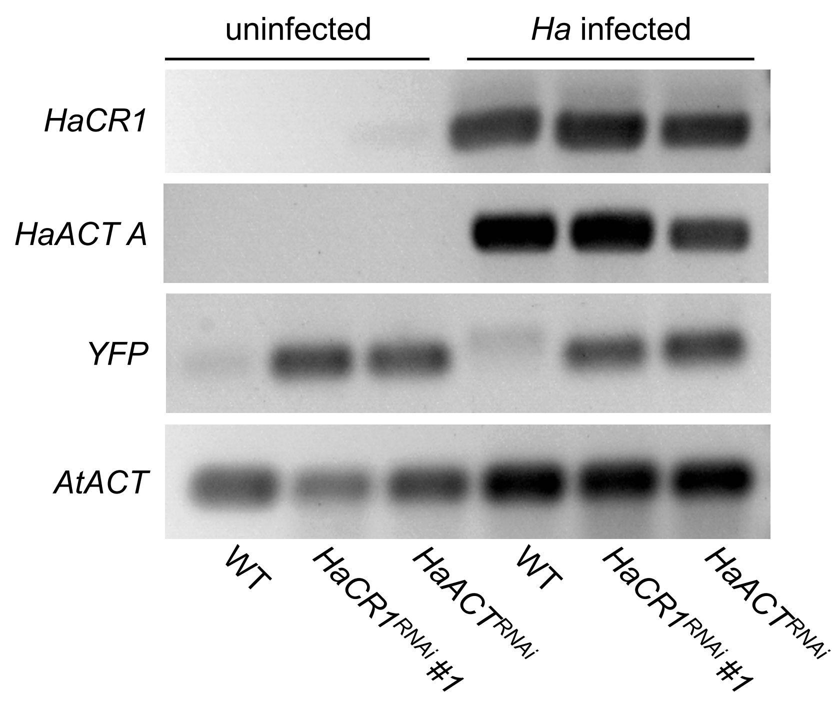

Figure S6: Validation of RT-PCR primers to assess *H. arabidopsidis* target gene expression in *A. thaliana* HIGS plants. RT-PCR of *H. arabidopsidis HaACT A* and *HaCR1* in mock-treated and *H. arabidopsidis*-infected *A. thaliana* WT and HIGS plants at 4 dpi. The HIGS construct did not generate any detectable PCR products with the *H. arabidopsidis*-specific target gene primers for *HaACT A* and *HaCR1* in non-infected plant samples. *YFP* expression was used as control for transgene expression in HIGS plants. *AtACT* was used to validate cDNA synthesis.

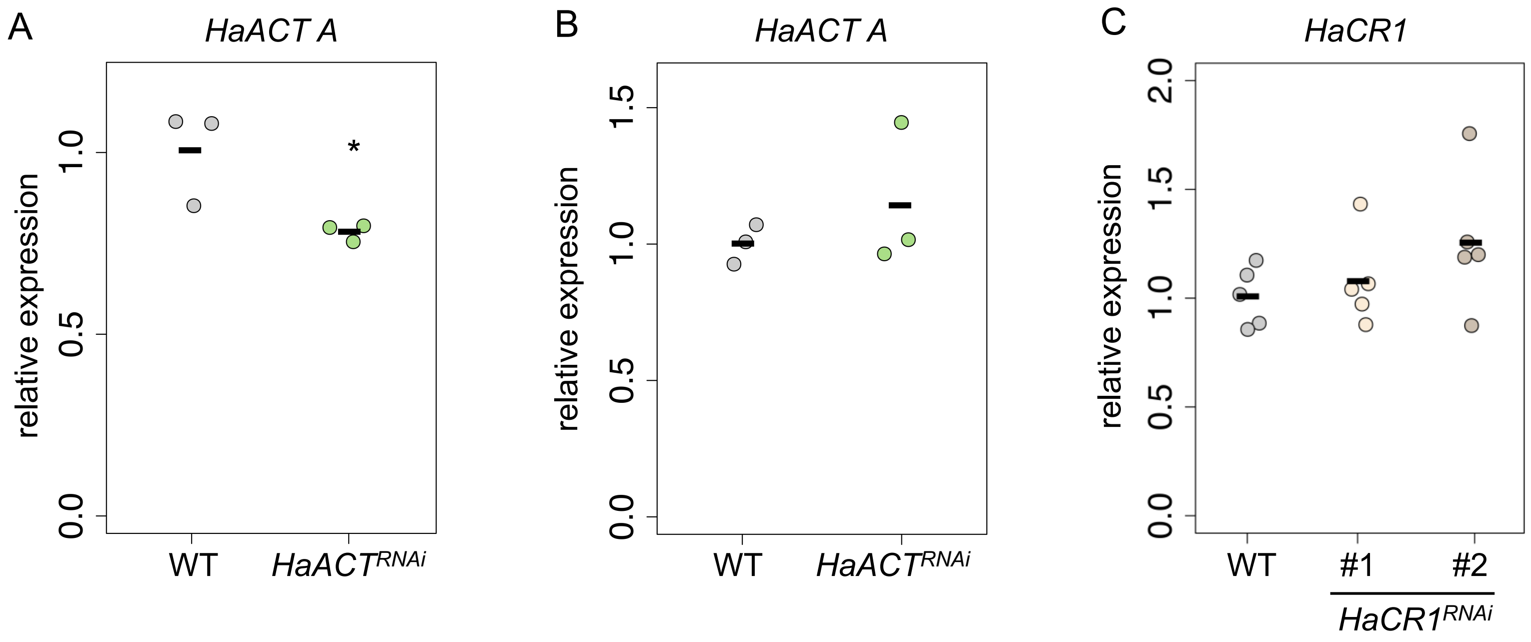

Figure S7: *H. arabidopsidis* target gene expression when infecting *A. thaliana* HIGS plants. A) Quantitative RT-PCR of *HaACT A* in *H. arabidopsidis*-infected *A. thaliana* WT and *HaACT*^{RNAi} plants at 4 dpi using *HaEF1α* as a reference genes. B) Quantitative RT-PCR of *HaACT A* in *H. arabidopsidis*-infected WT and corresponding HIGS plants at 7 dpi using *HaEF1α* as a reference gene. C) Quantitative RT-PCR of *HaCR1* in *H. arabidopsidis*-infected WT and two independent HIGS lines (#1, #2) at 7 dpi using *HaEF1α* and *HaWS021* as reference genes. The bars indicate the average of three biological replicates each comprising six to eight leaves. Asterisks indicate significant difference by student's t-test with $p \leq 0.05$.

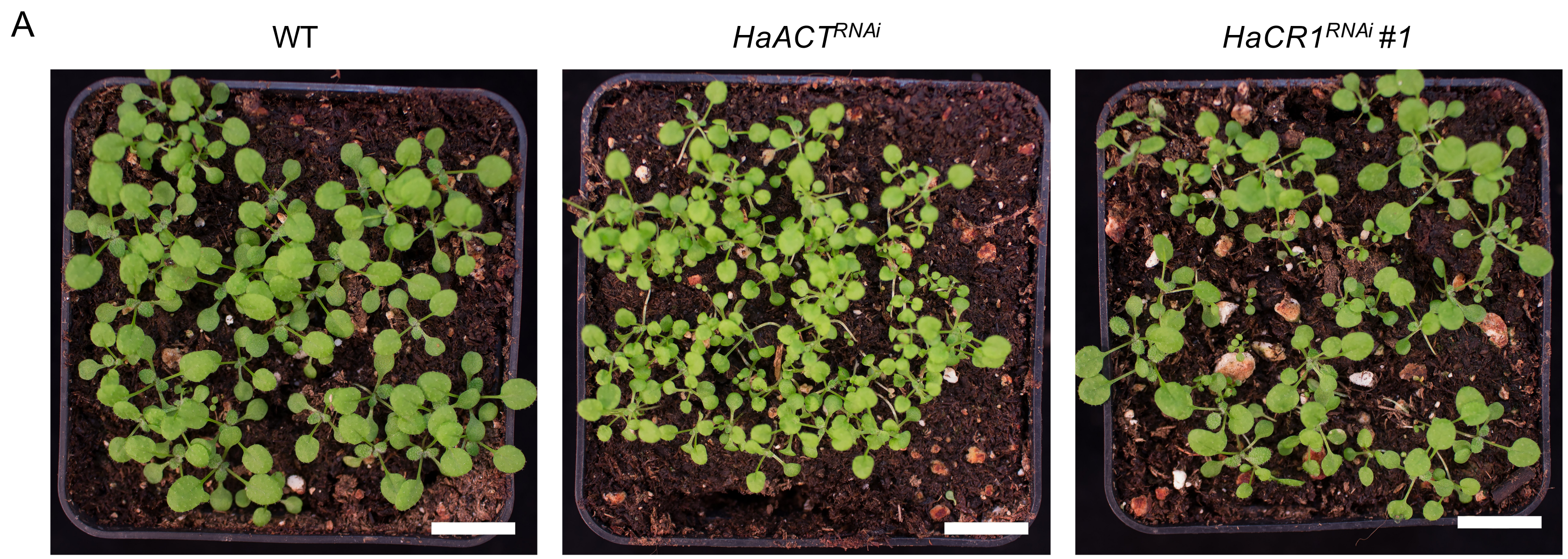

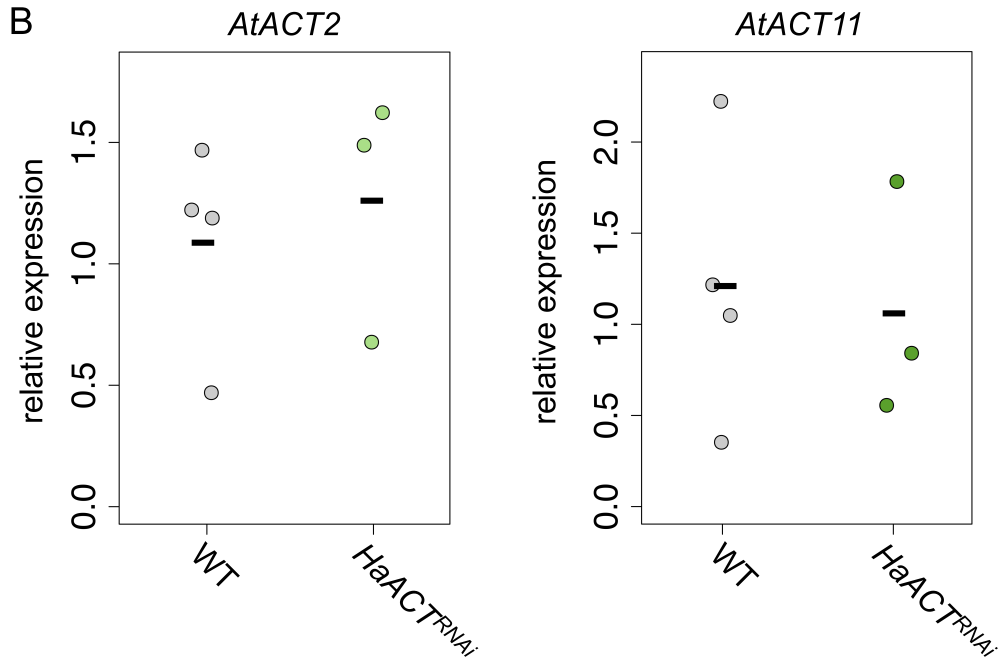

Figure S8: Growth phenotype of 14-days old *A. thaliana* WT, *HaCR1^{RNAi}* or *HaACT^{RNAi}* seedlings. A) Non-transgenic plants were removed beforehand taking pictures from the *HaCR1^{RNAi}* or *HaACT^{RNAi}* pots by selecting plants expressing a YFP transformation reporter with a fluorescence stereomicroscope. Scale bars represents 1 cm. B) The expression of the two closest *A. thaliana* paralogs of *HaACT A*, *AtACT2* and *AtACT11*, was determined by qRT-PCR using *AtTUB* and *AtUBQ10* as reference genes. The *HaACT^{RNAi}* construct did not alter the expression of *AtACT2* and *AtACT11* at 4 dpi.

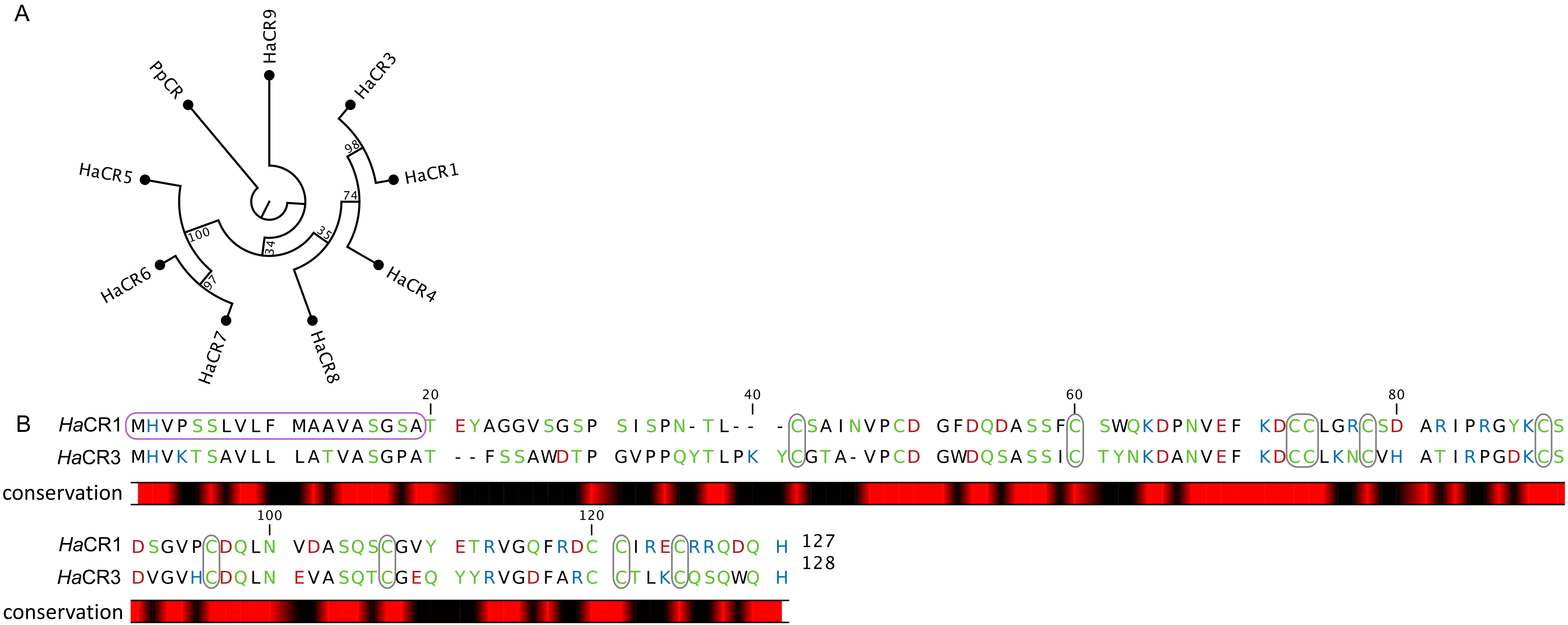

Figure S9: The *Ha*CR family in the *H. arabidopsidis* strain Noco2. A) Phylogenetic tree of *Ha*CRs based on the amino acid sequence. *Pp*CR from *P. parasitica* (gene ID: F443_03861) was used as an outgroup to root the tree. The numbers show bootstrap values of 100 bootstraps. B) Protein sequence alignment between *Ha*CR1 and its closest homolog *Ha*CR3. The signal peptide of *Ha*CR1 is highlighted within the purple box, the cysteine residues with grey boxes. Sequence conservation is displayed in red in the panel below the alignment.

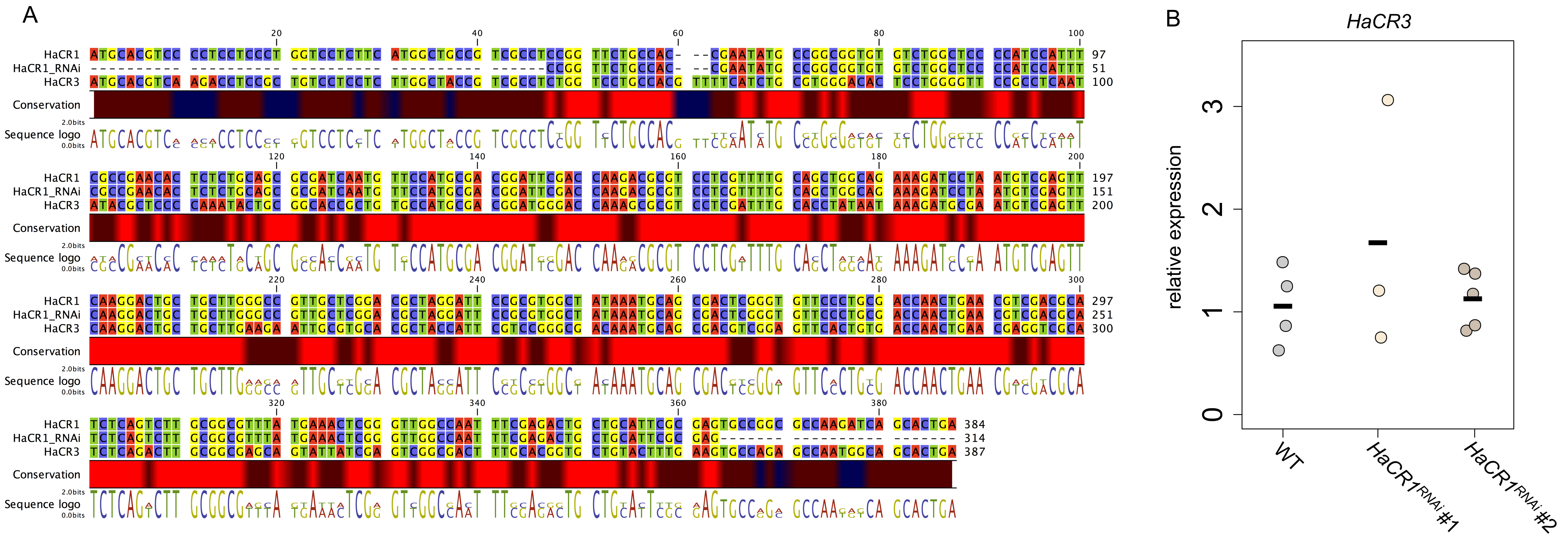

Figure S10: *HaCR3* expression was not suppressed during infection of *HaCR1^{RNAi}* plants. A) DNA Sequence alignment of the coding sequences of *HaCR1* and *HaCR3* with the sequence of the *HaCR1^{RNAi}* construct. B) Quantitative RT-PCR of *HaCR3* in *A. thaliana* WT and *HaCR1^{RNAi}* plants at 4 dpi using ≥ three biological replicates. *HaEF1α* and *HaWS021* were used as reference genes.

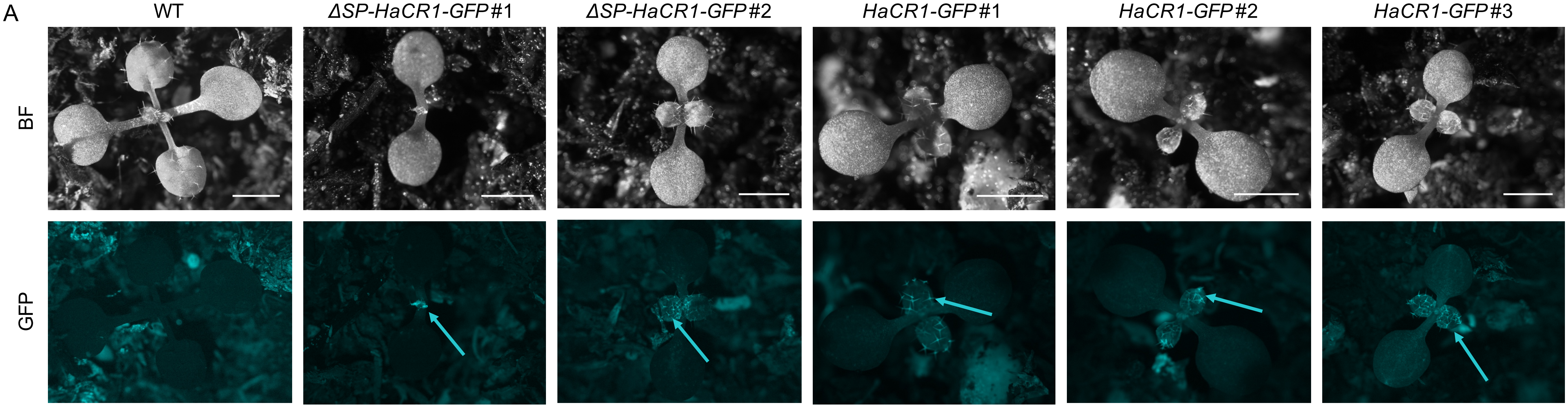

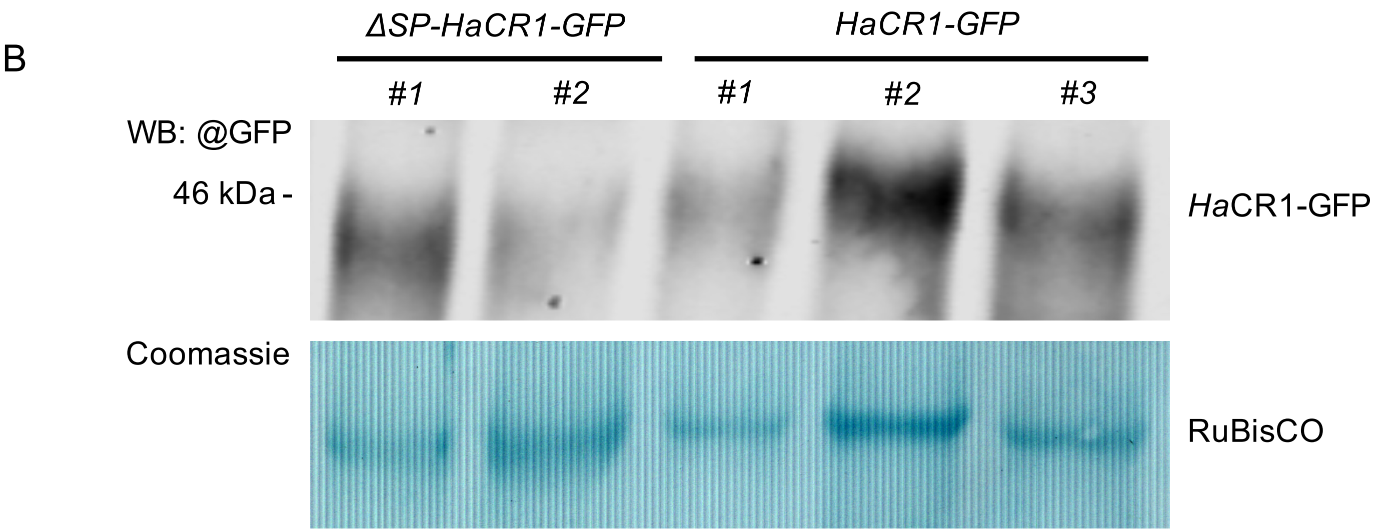

Figure S11: *A. thaliana* seedlings of individual transformation lines expressing *Ha*CR1-GFP or ΔSP-*Ha*CR1-GFP. A) GFP expression (teal arrow) was detected mainly in young leaves, but not in cotyledons of individual transformation lines (#) using a fluorescence stereomicroscope. The scale bars present 2 mm. B) Western blot was performed using an anti-GFP antibody to confirm *Ha*CR1-GFP (40.6 kDa) or ΔSP-*Ha*CR1-GFP (38.7 kDa) fusion proteins, with Coomassie staining of RuBisCO served as a loading control.

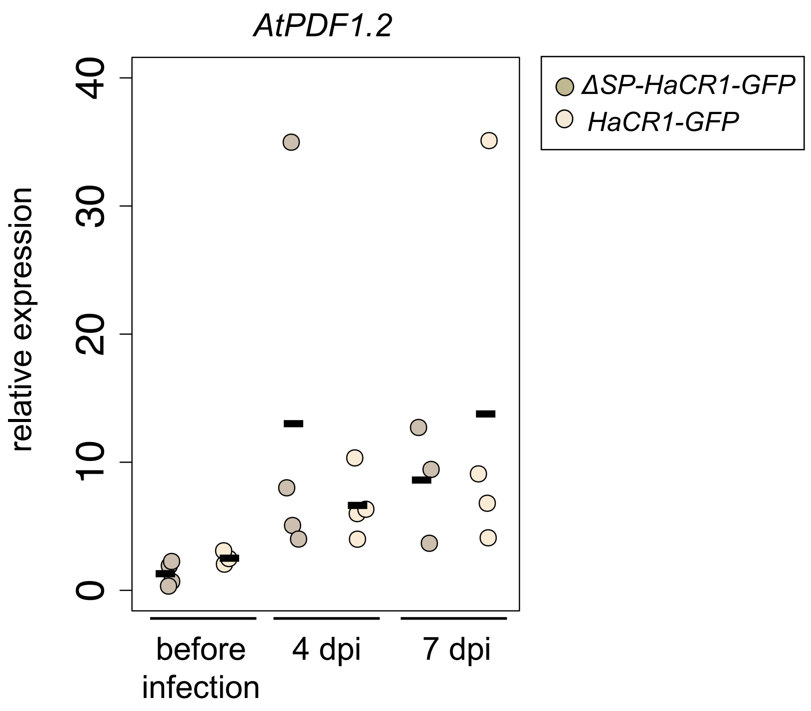

Figure S12: Expression of *AtPDF1.2* was not different when comparing Δ*SP-HaCR1-GFP* or *HaCR1-GFP* expressing *A. thaliana* seedlings upon *H. arabidopsidis* infection. *AtPDF1.2* expression was determined by qRT-PCR using *AtACT2* and *AtTUB* as reference genes.

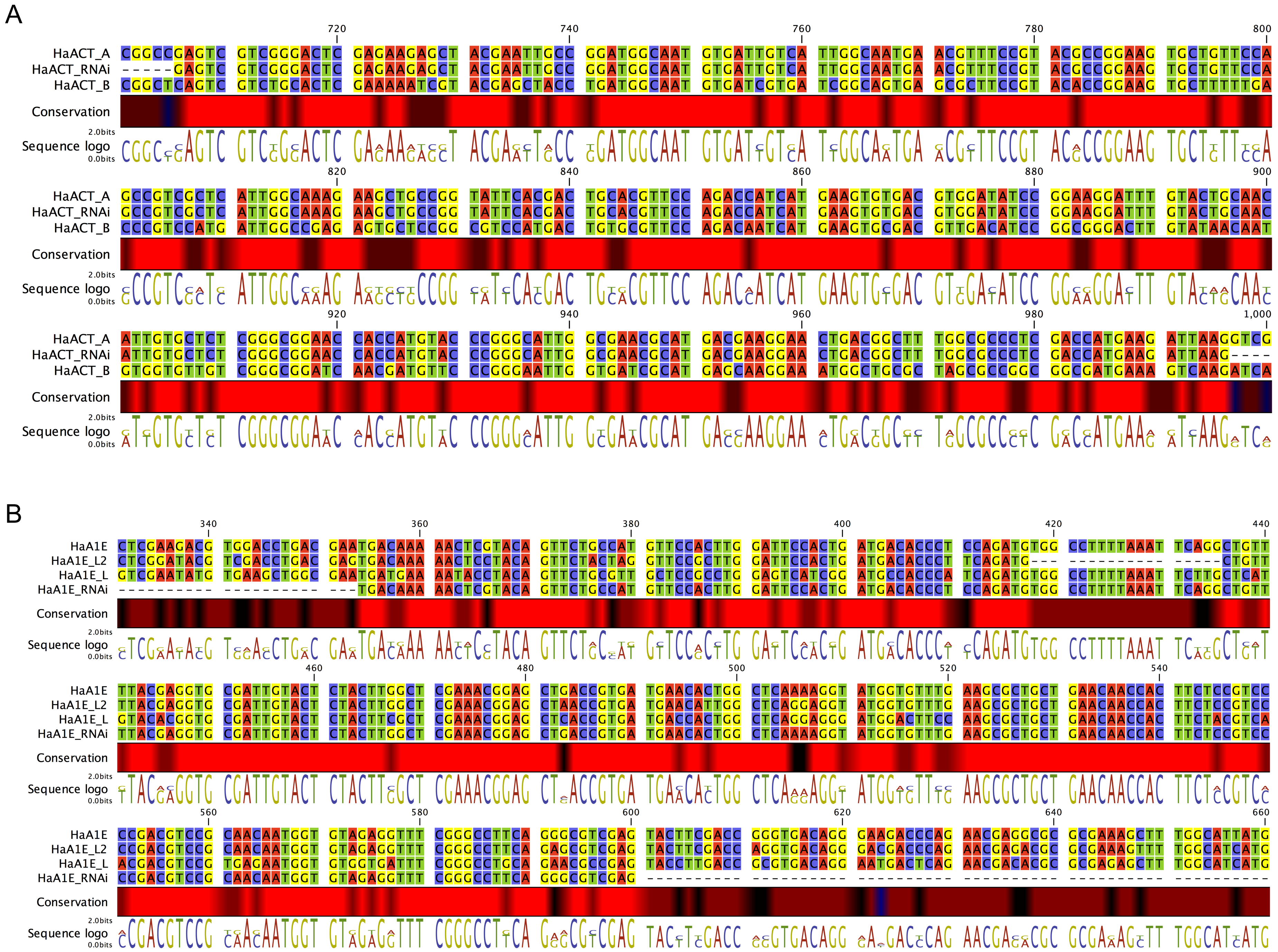

Figure S13: DNA sequence alignment of the target gene and closest paralogs at the HIGS target site. A) Sequence alignment of *HaACT A* with *HaACT B*. B) Sequence alignment of *HaA1E* with the two paralogs *HaA1EL* and *HaA1EL2*. The numbers above the alignments in A) and B) are showing the position in the target transcript.
